# RPL22L1, a novel candidate oncogene promotes temozolomide resistance by activating STAT3 in glioblastoma

**DOI:** 10.1038/s41419-023-06156-6

**Published:** 2023-11-20

**Authors:** Yunping Chen, Yu Mu, Qing Guan, Chenlong Li, Yangong Zhang, Yinzhi Xu, Chong Zhou, Ying Guo, Yanan Ma, Meiqi Zhao, Guohua Ji, Peng Liu, Donglin Sun, Haiming Sun, Nan Wu, Yan Jin

**Affiliations:** 1 Laboratory of Medical Genetics, Harbin Medical University, Harbin, 150081 China; 2 Key laboratory of preservation of human genetic resources and disease control in China (Harbin Medical University), Ministry of Education, Harbin, 150081 China; 3 College of Sports and Human Sciences, Harbin Sport University, Harbin, 150008 China; 4 Department of Neurosurgery, Harbin Medical University Cancer Hospital, Harbin, 150001 China; 5 Department of Neurosurgery, The Second Affiliated Hospital of Harbin Medical University, Harbin, 150086 China

**Keywords:** CNS cancer, Diagnostic markers

## Abstract

Aggressiveness and drug resistance are major challenges in the clinical treatment of glioblastoma (GBM). Our previously research reported a novel candidate oncogene ribosomal protein L22 like 1 (RPL22L1). The aim of this study was to elucidate the potential role and mechanism of RPL22L1 in progression and temozolomide (TMZ) resistance of GBM. Online database, tissue microarrays and clinical tissue specimens were used to evaluate the expression and clinical implication of RPL22L1 in GBM. We performed cell function assays, orthotopic and subcutaneous xenograft tumor models to evaluate the effects and molecular mechanisms of RPL22L1 on GBM. RPL22L1 expression was significantly upregulated in GBM and associated with poorer prognosis. RPL22L1 overexpression enhanced GBM cell proliferation, migration, invasion, TMZ resistance and tumorigenicity, which could be reduced by RPL22L1 knockdown. Further, we found RPL22L1 promoted mesenchymal phenotype of GBM and the impact of these effects was closely related to EGFR/STAT3 pathway. Importantly, we observed that STAT3 specific inhibitor (Stattic) significantly inhibited the malignant functions of RPL22L1, especially on TMZ resistance. RPL22L1 overexpressed increased combination drug sensitive of Stattic and TMZ both in vitro and in vivo. Moreover, Stattic effectively restored the sensitive of RPL22L1 induced TMZ resistance in vitro and in vivo. Our study identified a novel candidate oncogene RPL22L1 which promoted the GBM malignancy through STAT3 pathway. And we highlighted that Stattic combined with TMZ therapy might be an effective treatment strategy in RPL22L1 high-expressed GBM patients.

## Introduction

Glioblastoma (GBM) is the most common aggressive primary brain cancer in adults, with a median survival of only 14.6 months [1, 2]. Currently, GBM is mainly treated with surgical resection, radiotherapy and temozolomide (TMZ) chemotherapy as first-line therapy [3]. Due to the rapid development, strong tumor heterogeneity, treatment resistance and recurrence, the prognosis of GBM patients is extremely dismal [4]. Presently, GBM still needs more effective targets and biomarkers for precision treatment. Therefore, it is urgent to reveal the molecular mechanism of the pathogenesis and development of GBM, and identify effective biomarkers to provide theoretical basis for the treatment.

Epidermal growth factor receptor (EGFR) is one of the main therapeutic targets of GBM. Changes in EGFR are found in approximately 60% of GBM, which triggers tumor occurrence, development and TMZ resistance [5]. Whereas, due to the heterogeneity of GBM, targeted EGFR therapy is ineffective, partly because of the increase in epithelial-mesenchymal transition (EMT). In addition to EGFR, there are many other molecular modulators contributing to GBM malignancy and therapy resistance need to be further explored. For example, signal transducer and activator of transcription 3 (STAT3) is a vital pathogenic factor of GBM and can be specifically inhibited by Stattic [6]. The activation of STAT3 is also closely related to the malignant degree and poor prognosis of GBM [7]. But the application of STAT3 in the treatment of GBM still needs to be further explored.

Our team previously identified the ribosomal L22 like 1 (RPL22L1) was a candidate oncogene in ovarian cancer, which could promote metastasis by inducing EMT [8]. In addition, studies have shown that RPL22L1 was elevated in human prostate cancer tissues and promoted the proliferation and invasion of prostate cancer cells [9]. Furthermore, RPL22L1 was significantly elevated in colorectal cancer, which was related to poor prognosis and 5-fluorouracil resistance [10]. Up to now, the role of RPL22L1 in GBM has not been reported yet. In this study, we focused on the function and mechanism of RPL22L1 in GBM, thus providing a new potential GBM biomarker.

## Results

### RPL22L1 is highly expressed in GBM and related to poor prognosis

**Fig. 1:**
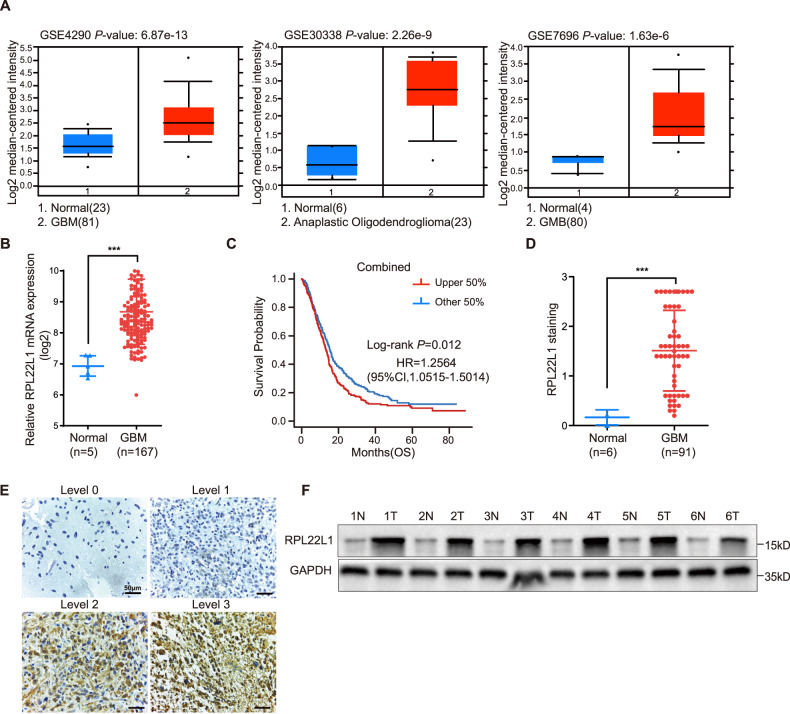
RPL22L1 is elevated in GBM patients and associated with poor prognosis of GBM. **A** RPL22L1 mRNA levels in GBM tissues were analyzed in GEO datasets through Oncomine platform (GSE4290, GSE30338, GSE7696, https://www.oncomine.org). **B** RPL22L1 mRNA was compared between normal brain tissues (*n* = 5) and GBM tissues (*n* = 167) in the TCGA dataset. Data were presented as the mean ± SD, ***P < 0.001, Student’s *t* test. **C** Kaplan-Meier survival analysis according to RPL22L1 mRNA levels in GBM patients (*n* = 882, Log-rank test, *P* = 0.012 HR = 1.2564, 95%CI, 1.0515–1.5014) from GEO datasets (GSE42669, GSE4412, GSE7696), CGGAarry, CGGAseq and TCGA combined. **D** IHC staining statistics of RPL22L1 protein in normal brain tissues (*n* = 6) and GBM tissues (*n* = 91) in TMAs. The staining score = staining intensity (0–3) × staining area ratio (0–100%). ***P < 0.001, Student’s *t* test. **E** Representative images of four levels of RPL22L1 IHC staining in GBM tissues (magnification×400, scale bar = 50 μm). **F** The expressions of RPL22L1 protein in clinical GBM tissues (T) and paired adjacent tissues (N) were detected by Western Blot (*n* = 6), GAPDH was the internal reference.

GEO and TCGA RNA-seq expression datasets were used to evaluate the mRNA expression of RPL22L1, results showed it was significantly higher in GBM and oligodendroglioma tissues than that in normal brain tissues (Fig. 1A, B). Importantly, GBM patients with high RPL22L1 expression had a shorter survival time and worse prognosis in TCGA, GEO and CGGA combined datasets (Fig. 1C).

Consistent with the datasets, the expression level of RPL22L1 protein in GBM was significantly higher than that in normal brain tissues confirmed by immunohistochemistry (IHC) (Fig. 1D). The RPL22L1 protein expression levels were classified into 0–3 levels according to the staining intensity (Fig. 1E). Among 179 cases of glioma tissues, 115 (64.2%) cases had high expression level of RPL22L1 protein (staining intensity 2–3 levels). Among 91 cases of GBM tissues, 67 (73.6%) cases had high expression level of RPL22L1 protein (Table 1). Moreover, Western Blot showed that the expression level of RPL22L1 protein in GBM tissues was significantly higher than that in adjacent tissues (Fig. 1F).

**Table 1:** The protein expression of RPL22L1 in glioma tissue microarray.

Tissues	All cases	RPL22L1 expression -low (0–1 levels)	RPL22L1 expression- high (2–3 levels)
Normal	6	6 (100.0%)	0 (0.0%)
Glioma	179	64 (35.8%)	115 (64.2%)
GBM	91	24 (26.4%)	67 (73.6%)

Relationship between protein expression and clinicopathological characteristics was evaluated, and it was found that the expression level of RPL22L1 was positively correlated with the pathological stage of patients (*P* = 0.035) (Table 2). These results indicate that the expression of RPL22L1 in GBM is increased compared with normal brain tissues, which is closely related to the poor prognosis of GBM patients.

**Table 2:** Correlation between RPL22L1 expression and clinicopathologic features of gliomas.

Variable	All cases	RPL22L1 expression-low	RPL22L1 expression-high	*P*-value
Gender				0.492
Male	112	29 (25.9%)	83 (74.1%)	
Female	67	21 (31.3%)	46 (68.7%)	
Age				0.484
≤56	115	30 (26.1%)	85 (73.9%)	
>56	61	19 (31.1%)	42 (68.9%)	
Stage				0.035^a^
II-III	74	32 (43.2%)	42 (56.8%)	
IV	100	27 (27.0%)	73 (73.0%)	

^a^*P* = 0.035, Fisher’s exact test was used for statistical analysis. Missing age and clinical grade information for individual patients.

### RPL22L1 enhances the malignant function of GBM cells in vitro and in vivo

**Fig. 2:**
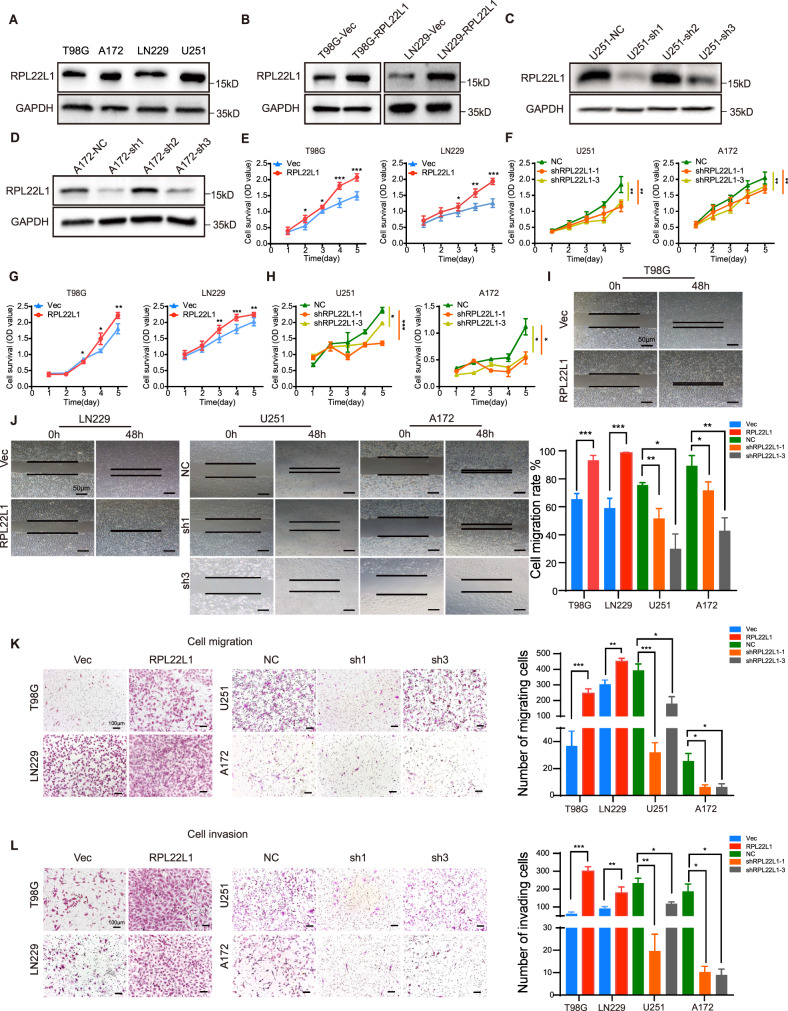
RPL22L1 promotes GBM cell proliferation, migration and invasion in vitro. **A** The expressions of endogenous RPL22L1 protein in T98G, A172, LN229 and U251 were detected by Western Blot, GAPDH was used as the internal reference. **B** T98G and LN229 cell lines were infected with RPL22L1 lentivirus, vector was the control group. After 72 h, Western Blot was used to detect the expressions of RPL22L1 protein. **C**, **D** RPL22L1 expressions were efficiently knocked down by two targeted shRNAs (sh1 and sh3) in U251 and A172 cell lines detected by Western Blot, NC served as negative control. **E**, **F** The effects of RPL22L1 on the viability of GBM cells were detected by MTS assay (*n* = 6). **G**, **H** The effects of RPL22L1 on the viability of GBM cells were detected by CCK8 assay (*n* = 6). **I**, **J** Wound healing assay was used to detect the cell migration abilities of cells (magnification×40, scale bar = 50 μm). **K**, **L** Transwell migration and invasion assays were performed to examine the effects of RPL22L1 on migration (**K**) and invasion (**L**) of GBM cells (left, magnification×100, scale bar = 100 μm). All data were shown as mean ± SD of three independent experiments (*P < 0.05; **P < 0.01; ***P < 0.001, Student’s *t* test).

To further investigate the effects of RPL22L1 on the biological function of GBM cells, we established cell models with different RPL22L1 expression levels. According to the endogenous RPL22L1 expressions (Fig. 2A), two stable overexpression GBM cell lines and the control were established (T98G-RPL22L1/Vec and LN229-RPL22L1/Vec, Fig. 2B). Three short hairpin RNA (shRNA) sequences were used to knock down RPL22L1, among which sh1 and sh3 interference were the more successful and were used to establish stable knockdown cell lines and the negative control (U251-shRPL22L1-1, U251-shRPL22L1-3/NC, A172-shRPL22L1-1, A172-shRPL22L1-3/NC, Fig. 2C, D).

Cell proliferation curve (MTS and CCK8), wound healing, migration and invasion assays were used to detect the effects of RPL22L1 on these related cell functions. Overexpression of RPL22L1 significantly promoted the proliferation (Fig. 2E–H), migration (Fig. 2I–K) and invasion (Fig. 2L) of GBM cells. On the contrary, RPL22L1 knockdown significantly reduced these abilities. These data suggest that RPL22L1 enhances the proliferation, migration and invasion ability of GBM cells in vitro.

**Fig. 3:**
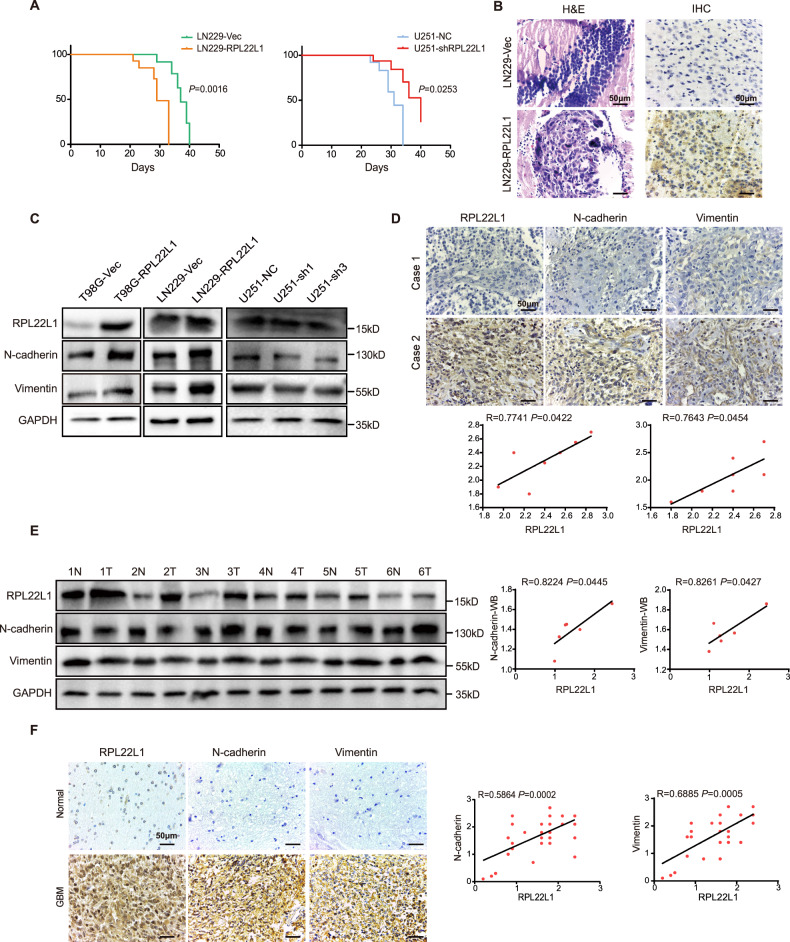
RPL22L1 induces mesenchymal phenotype in GBM. **A** Kaplan-Meier overall survival analysis of xenograft tumor in nude mice with Log-rank test, *P*-values indicated (*n* = 10). **B** Representative H&E and IHC RPL22L1 staining images (magnification × 400, scale bar = 50 μm) in section of xenograft tumors induced with LN229-RPL22L1 and control cells were shown. **C** The expressions of N-cadherin and Vimentin in GBM cells were detected by Western Blot, GAPDH was used as the internal control. **D** Representative images of IHC staining for clinical GBM tissues showed the expressions of RPL22L1, N-cadherin and Vimentin (left, magnification × 400, scale bar = 50 μm). Case 1: GBM with low RPL22L1 expression; Case 2: GBM with high RPL22L1 expression. According to the staining scores to analyze the correlations between RPL22L1 and N-cadherin, Vimentin (right, Pearson correlation analysis, *n* = 7). **E** Western Blot was used to analyze the expressions of N-cadherin and Vimentin in clinical GBM tissues and paired adjacent tissues (left, *n* = 6). Protein gray scale analysis of the correlations between RPL22L1, N-cadherin and Vimentin (right, Pearson correlation analysis, *n* = 6). **F** IHC staining of RPL22L1, N-cadherin and Vimentin in normal brain tissues and GBM tissues in TMAs (left, magnification × 400, scale bar = 50 μm). According to the staining scores to analyze the correlations between RPL22L1 and N-cadherin, Vimentin expressions (right, Pearson correlation analysis) in normal brain tissues (*n* = 3) and GBM brain tissues (*n* = 32).

To examine the effects of RPL22L1 on the GBM in vivo, we established mice GBM orthotopic xenograft models bearing using two paired cell lines. Mice bearing GBM derived from LN229-RPL22L1 and U251-NC exhibited significantly poorer survival (Fig. 3A). Formation of GBM was confirmed with H&E staining, the tumor cell nucleus was dense, deeply stained, pyknotic and fence-like. IHC detection showed that the level of RPL22L1 increased after overexpression of RPL22L1 (Fig. 3B). These results show that RPL22L1 enhances malignant functions of GBM cells.

### RPL22L1 promotes mesenchymal phenotype of GBM

EMT is a significant process regulating GBM invasion and progression [11]. Since RPL22L1 enhanced malignant progression of GBM cells, we further explored whether it participated in the EMT progress of GBM. Western Blot was used to detect the EMT-related markers in cell models. The results suggested that overexpression of RPL22L1 up-regulated the expressions of N-cadherin, Vimentin and it was further verified by immunofluorescence (IF) staining (Supplementary Fig. S1A). On the contrary, knockdown RPL22L1 could reverse these expressions (Fig. 3C). Influences on the E-cadherin, β-catenin and other related factors such as α-SMA, Snail2 and Twist1 were also observed (Supplementary Fig. S1B).

Further, we confirmed the relationships between RPL22L1 and these two mesenchymal markers in clinical GBM tissues. IHC staining of 7 clinical GBM pathological tissue sections confirmed these correlations. Statistical analysis of IHC staining area and staining intensity product scores showed positive correlations between RPL22L1 and N-cadherin (*R* = 0.7741, *P* = 0.0422), RPL22L1 and Vimentin (*R* = 0.7643, *P* = 0.0454) (Fig. 3D). Western Blot results also showed that GBM tissues had higher levels of N-cadherin and Vimentin than paired adjacent tissues. And there were significantly positive correlations between RPL22L1 and N-cadherin expression (*R* = 0.8224, *P* = 0.0445), RPL22L1 and Vimentin expression (*R* = 0.8261, *P* = 0.0427) in GBM tissues (Fig. 3E).

In addition, we also performed IHC staining on the tissue microarrays (TMAs) (3 normal brain tissues and 32 GBM brain tissues), and statistical analysis of IHC staining area and staining intensity product scores showed that, RPL22L1 was positively correlated with N-cadherin (*R* = 0.5864, *P* = 0.0002), RPL22L1 was positively correlated with Vimentin (*R* = 0.6885, *P* = 0.0005) (Fig. 3F). Overall, these data demonstrate that RPL22L1 promotes the mesenchymal phenotype to obtain malignant invasiveness of GBM.

### RPL22L1 activates the EGFR/STAT3 pathway to promote GBM malignancy

**Fig. 4:**
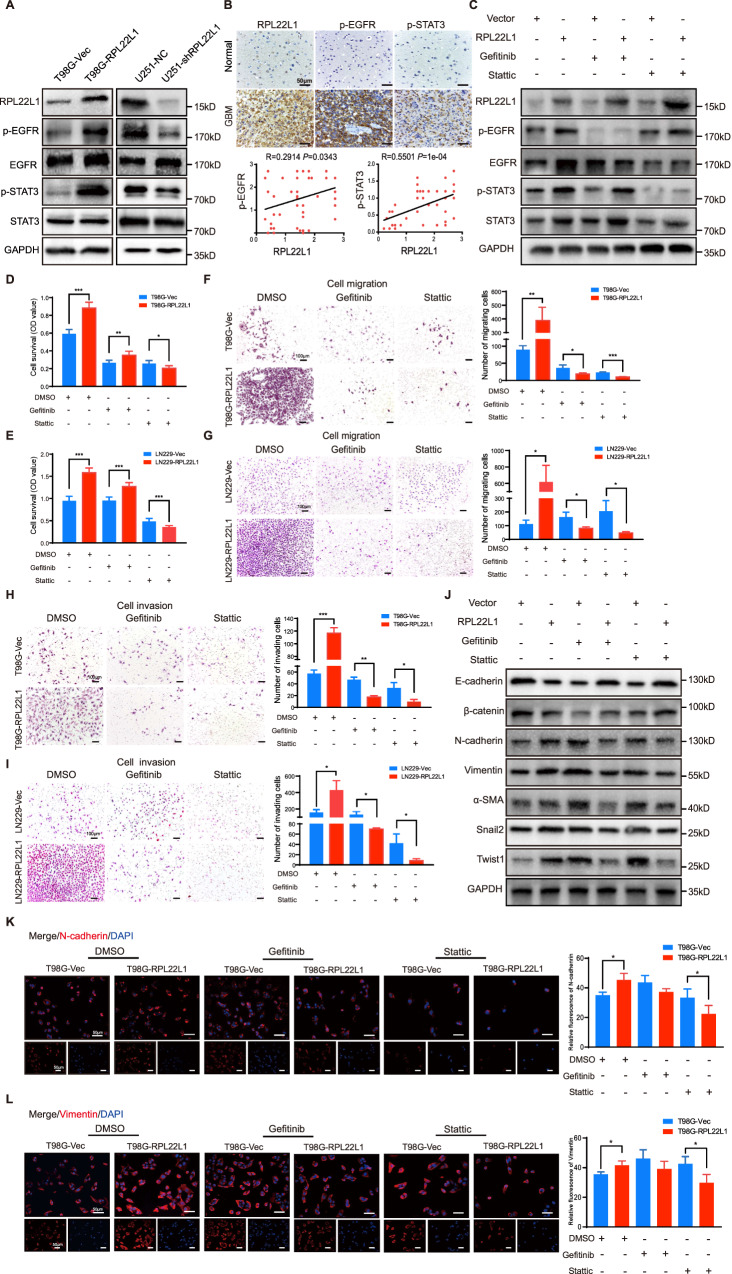
RPL22L1 facilitates the malignant progression of GBM and induces mesenchymal phenotype through the EGFR/STAT3 pathway. **A** Western Blot analysis of the effects of RPL22L1 on the protein levels of p-EGFR, EGFR, p-STAT3 and STAT3. **B** IHC staining of RPL22L1, p-EGFR and p-STAT3 in normal brain tissues and GBM brain tissues in TMAs (up, magnification×400, scale bar = 50 μm). According to the staining scores to analyze the correlations between RPL22L1 and p-EGFR, p-STAT3 protein expressions (down, Spearman correlation analysis, *n* = 53). **C**–**G** Cells were treated with Gefitinib (10 μmol/L) or Stattic (5 μmol/L) for 48 h. **C** Western Blot detected the expressions of RPL22L1, EGFR, p-EGFR, STAT3 and p-STAT3, GAPDH was as the internal control. **D**, **E** MTS assay and CCK8 assay detected the effects of Gefitinib or Stattic on the viability of T98G-RPL22L1/T98G-Vec cells (**D**) and LN229-RPL22L1/LN229-Vec cells (**E**). All data were shown as mean ± SD of three independent experiments (*P < 0.05, **P < 0.01; ***P < 0.001, Student’s *t* test). **F**, **G** Transwell migration assays detected the effects of Gefitinib or Stattic on the migration of T98G-RPL22L1/T98G-Vec cells (**F**) and LN229-RPL22L1/LN229-Vec cells (**G**). **H**, **I** Transwell invasion assays detected the effects of Gefitinib or Stattic on the invasion of T98G-RPL22L1/T98G-Vec cells (**H**) and LN229-RPL22L1/LN229-Vec cells (**I**). Representative images were shown (left, magnification×100, scale bar = 100 μm). All data were shown as mean ± SD of three independent experiments (right, *P < 0.05, **P < 0.01; ***P < 0.001, Student’s *t* test). **J** Western Blot was used to detect the expressions of E-cadherin, β-catenin, N-cadherin, Vimentin, α-SMA, Snail2 and Twist1 in T98G-RPL22L1/T98G-Vec cells, GAPDH was the internal control. **K**, **L** IF assay detected the expressions of N-cadherin (**K**) and Vimentin (**L**) protein (left, red: N-cadherin and Vimentin, blue: cell nucleus, magnification×200, scale bar = 50 μm). Statistics of relative IF value of Gefitinib and Stattic on the expressions of N-cadherin and Vimentin in T98G-RPL22L1/T98G-Vec cells. All data were shown as mean ± SD of three independent experiments (right, *P < 0.05, Student’s *t* test).

Activation of the EGFR/STAT3 pathway plays important role in GBM progression [12]. As detected by Western Blot in GBM cells overexpressing RPL22L1, p-EGFR and p-STAT3 were significantly elevated. On the contrary, they were obviously attenuated in GBM cells knocked down RPL22L1 (Fig. 4A). To detect the relationships between EGFR, STAT3 protein phosphorylation levels and RPL22L1 in GBM, IHC analysis was performed on TMAs. We observed positive correlations between p-EGFR and RPL22L1 (*R* = 0.2914, *P* = 0.0343), p-STAT3 and RPL22L1 (*R* = 0.5501, *P* = 1e-04) (Fig. 4B). In addition, these correlations were also detected in xenograft tumors (Supplementary Fig. S2A).

To further confirm whether RPL22L1 directly regulates EGFR/STAT3 pathway to participate in GBM progression, EGFR inhibitor-Gefitinib and STAT3 inhibitor-Stattic were used to treat the cell models. Western Blot was used to verify the inhibition effects (Fig. 4C). In function assays, Stattic could significantly reduce the viability of cells overexpressing RPL22L1, but Gefitinib couldn’t (Fig. 4D, E). Both Gefitinib and Stattic could significantly reduce the migration (Fig. 4F, G, Supplementary Fig. S2B) and invasion capabilities (Fig. 4H, I) of cells overexpressing RPL22L1. The increases of EMT-related factors N-cadherin, Vimentin, α-SMA, Snail2 and Twist1, the decreases of E-cadherin and β-catenin caused by RPL22L1 were inhibited by the two inhibitors (Fig. 4J). IF staining also confirmed that the two inhibitors inhibited the increases of N-cadherin and Vimentin induced by RPL22L1 (Fig. 4K, L). These results indicate that RPL22L1 enhances the mesenchymal phenotype and promotes GBM progression by activating EGFR/STAT3.

### RPL22L1 enhances TMZ resistance of GBM cells through STAT3

**Fig. 5:**
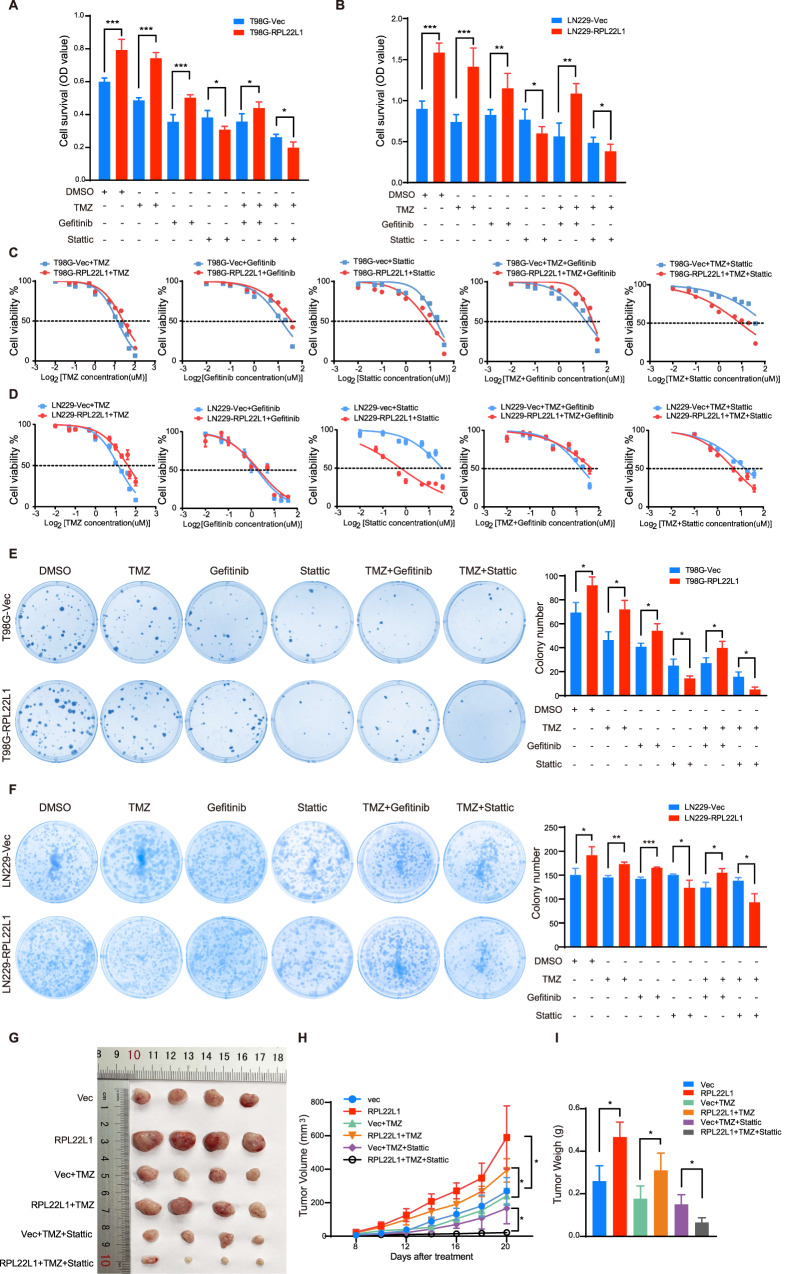
RPL22L1 enhances TMZ resistance and can be reversed by Stattic. **A–F** GBM cells were treated with TMZ (7.5 μmol/L), Gefitinib (10 μmol/L), Stattic (5 μmol/L), TMZ+Gefitinib, TMZ+Stattic for 48 h, DMSO was the negative control. **A,** **B** MTS experiment and CCK8 assay were performed to detect proliferation of T98G-RPL22L1/T98G-Vec cells (**A**) and LN229-RPL22L1/LN229-Vec cells (**B**). **C**, **D** T98G-RPL22L1/T98G-Vec cells (**C**) and LN229-RPL22L1/LN229-Vec cells (**D**) were treated at the specified concentration for 48 h and IC50 were determined by MTS and CCK8 methods. **E**, **F** Colony formation tests of T98G-RPL22L1/T98G-Vec cells (**E**) and LN229-RPL22L1/LN229-Vec cells (**F**). All data were shown as mean ± SD of three independent experiments (*P < 0.05, **P < 0.01, ***P < 0.001, Student’s *t* test). **G–I** LN229-RPL22L1/LN229-Vec cells were injected subcutaneously into the right shoulder of nude mice. One week later, intraperitoneal injection of TMZ (60 mg/kg), TMZ+Stattic (5 mg/kg) to nude mice bearing tumors for 13 days. After drug treatment, the subcutaneous tumors were taken, photographed (**G**) and weighed (**I**). The tumor size (**H**) was monitored every other day and statistics were performed (*n* = 4, *P < 0.05, Student’s *t* test).

EGFR and STAT3 play important roles in TMZ therapy in GBM and influence prognosis [13, 14]. Since RPL22L1 promotes the malignant of GBM by activating EGFR/STAT3, we further explore the role of this relationship in TMZ treatment. To assess the effects of RPL22L1 on the therapeutic effect of TMZ, TMZ, Gefitinib, Stattic, TMZ+Gefitinib, TMZ+Stattic were used separately to treat cell models overexpressing RPL22L1. After treatment with TMZ, the overexpression of RPL22L1 increased the GBM cell viability, indicating that RPL22L1 enhanced the TMZ resistance. The effect of RPL22L1 on cell survival could be reversed only after Stattic treatment (with or without TMZ, Fig. 5A, B).

Meanwhile, we examined the effects of RPL22L1 in GBM resistant cell lines. First, we analyzed the GSE131897 and GSE234762 datasets using GEO2R online analysis platform of NCBI and found that RPL22L1 was highly expressed in TMZ resistance cells (Supplementary Fig. S3A, B). Subsequently, we detected the expression levels of RPL22L1 in TMZ resistance cell line LN229R (Supplementary Fig. S4A, B). And we observed the changes of IC50 by knocking down RPL22L1 in LN229R. The results show that RPL22L1 promotes TMZ resistance in LN229R, and conversely, cells are sensitive to TMZ after RPL22L1 knockdown (Supplementary Fig. S4C, D). Besides, overexpression of RPL22L1 in GBM cells led to adverse reactions to TMZ, as shown by increased IC50. However, Stattic significantly reduced the IC50 of TMZ and showed a synergy in inhibiting the proliferation in RPL22L1-overexpressed cells (Fig. 5C, D). The cell colony formation was more weakened in RPL22L1 overexpressed cells only when Stattic treated and combined Stattic and TMZ treatment (Fig. 5E, F). In addition, we analyzed the expressions of RPL22L1 mRNA in four GBM cell lines from The Human Protein Atlas (https://www.proteinatlas.org/) (Supplementary Fig. S5). These results demonstrate that RPL22L1 promotes cell proliferation and TMZ resistance mainly dependent on STAT3 but not EGFR.

In order to evaluate the effects of RPL22L1-STAT3 on the treatment of TMZ in vivo, we constructed subcutaneous xenografts and treated them with different drugs. After subcutaneous tumorigenesis, normal saline/TMZ/TMZ combined with Stattic were injected intraperitoneally in each group for 13 days. The results showed that RPL22L1 enhanced the subcutaneous tumorigenicity of GBM cells, and still showed a larger tumor volume in TMZ treatment. But after TMZ treatment combined with Stattic, the volume and weight of the tumors were significantly reduced in those formed by the overexpressing RPL22L1 GBM cells (Fig. 5G–I). The results indicate that RPL22L1 enhances the tumorigenic ability of GBM cells, and this ability can’t be inhibited by TMZ, but can be reversed after combination with Stattic.

**Fig. 6:**
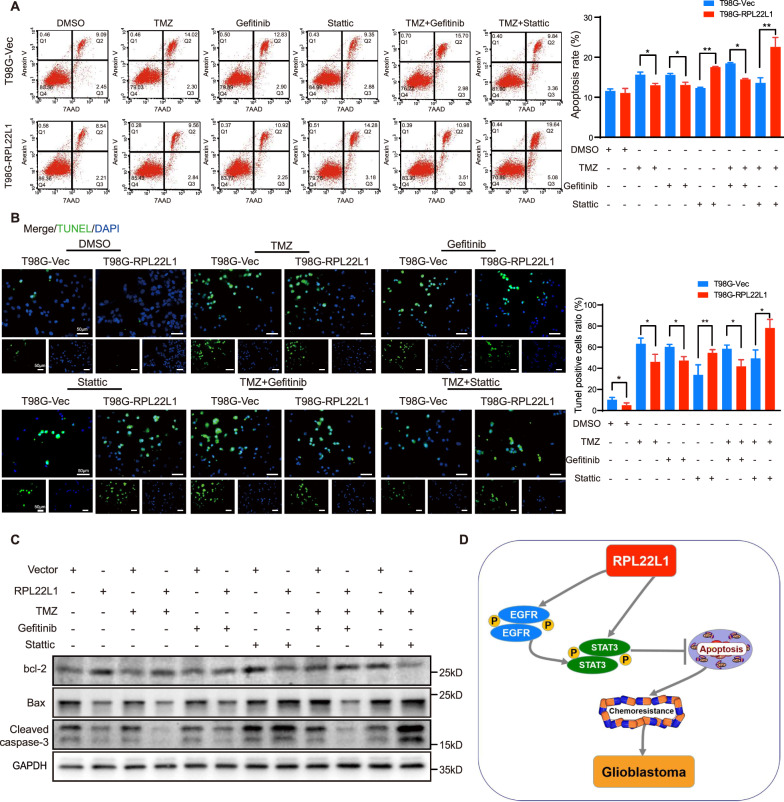
RPL22L1 inhibits GBM cell apoptosis through the STAT3 pathway. **A–C** T98G-RPL22L1/T98G-Vec cells were treated with TMZ (7.5 μmol/L), Gefitinib (10 μmol/L), Stattic (5 μmol/L), TMZ+Gefitinib, TMZ+Stattic for 48 h, DMSO was the negative control. **A** The cell apoptosis assay was performed with flow cytometry in T98G-RPL22L1/T98G-Vec cells. The histogram displayed the statistics of apoptosis assay, respectively. **B** The TUNEL assay of T98G-RPL22L1/T98G-Vec cells (left, green: TUNEL, blue: cell nucleus, magnification × 200, scale bar = 50 μm). All data were shown as mean ± SD of three independent experiments (right, *P < 0.05, **P < 0.01, Student’s *t* test). **C** Western Blot was used to detect the protein expressions of bcl-2, Bax and Cleaved-caspase-3, GAPDH was used as the internal control. **D** The mechanism scheme of RPL22L1 in GBM.

To further investigate these impacts, we detected cell apoptosis through flow cytometry and TUNEL assays. The apoptosis rates of cells overexpressing RPL22L1 were enhanced only after Stattic treatment especially combined with TMZ (Fig. 6A, B). The expressions of apoptosis related factors were detected by Western Blot. Only treated with Stattic and combined application with TMZ, the expressions of pro-apoptotic factors Cleaved-caspase-3 and Bax were increased in cells overexpressing RPL22L1, the expression of inhibitory apoptosis factor bcl-2 was decreased (Fig. 6C). Accordingly, these results suggest that although RPL22L1 promotes malignant progression of GBM through EGFR/STAT3 activation, the TMZ resistance caused by RPL22L1 is mainly result in the inhibition of apoptosis induced by STAT3 activation (Fig. 6D).

## Discussion

GBM is the most aggressive and common malignant glioma with poor prognosis due to the highly invasive, recurrence and drug resistance [15, 16]. Elucidating the intrinsic molecular mechanisms responsible for GBM progression is critical for identifying effective therapeutic approaches. In this study, we demonstrated that RPL22L1 is a candidate biomarker of GBM and an indicator of Stattic combined therapy. RPL22L1 is capable of modulating EGFR-STAT3 to enhance invasiveness in concert with mesenchymal changes, and cause TMZ resistance by activating STAT3.

Invasiveness is one of the most important characteristics leading to poor prognosis of GBM. Diffuse tumor invasion into adjacent brain restricts curative resection and the effective implementation of chemotherapy and radiotherapy, which is also considered to be the main cause of recurrence [17, 18]. The oncogenic activity of RPL22L1 has been predominant described outside the central nervous system, which is related to cell invasion, metastasis, EMT and drug resistance. Here, we found RPL22L1 is specifically highly expressed in GBM and is associated with poor prognosis and malignant progression. RPL22L1 can motivate the invasiveness and malignant progression of GBM cells in vivo and in vitro. Therefore, it is of potential clinical significance to clarify its role and mechanism in GBM.

EMT has emerged as a key regulator of the invasive state, which is closely related not only to invasiveness but also to drug resistance [19]. In terms of epithelial and mesenchymal phenotype, the majority of GBM samples analyzed have a profile more mesenchymal than epithelial [20]. The mesenchymal change, analogous to carcinoma EMT may contribute broadly to GBM malignancy. Various cytokines, such as EGFR and STAT3, initially trigger mesenchymal transition in GBM [21, 22]. In this study, we further found RPL22L1 could promote the expression of clinically relevant mesenchymal molecular N-cadherin and Vimentin, activate EGFR and STAT3. Inhibitor of EGFR (Gefitinib) or STAT3 (Stattic) could inhibit the enhanced invasiveness and the expressions of N-cadherin and Vimentin induced by RPL22L1, but only Stattic had an effect on the enhanced proliferation. In our study, Gefitinib downregulated p-EGFR to the levels that were similar between the vector- and RPL22L1-carrying cells. However, unlike the case with the control cells, Gefitinib failed to downregulate p-STAT3 in RPL22L1 overexpression cells. This indicates that RPL22L1 activates STAT3 in EGFR-independent manner, and RPL22L1 overexpression is more dependent on STAT3. Together these studies demonstrate that RPL22L1 is capable of modulating EGFR-STAT3 signaling, thereby promoting invasiveness function and mesenchymal phenotype of GBM.

EGFR-STAT3 activation is one of the characteristics of GBM, which is associated with mesenchymal transition and TMZ resistance [23]. TMZ is an oral DNA alkylating agent for the first-line treatment of GBM, but more than 50% of the treatment failed due to drug resistance [24, 25]. Targeting of the molecules responsible for TMZ resistance is crucial to block GBM evasion of therapeutic responses. As we found EGFR/STAT3 plays an important role in RPL22L1 promoting invasiveness but only STAT3 mediates the proliferation, it is speculated that STAT3 may play a different role in the survival related function of RPL22L1. After further exploring the role and mechanism of RPL22L1 in TMZ treatment, we found only Stattic combination could reverse the TMZ resistance and trigger apoptosis in the case of RPL22L1 overexpression.

STAT3 is a central hub associated with GBM progression and one of the master regulators of mesenchymal transformation [26]. In this study, we found that GBM cells overexpressing RPL22L1 activate the STAT3 pathway, making these cells dependent on the STAT3 pathway. As an apoptosis-inhibiting gene, directly downstream of STAT3, bcl-2 has a significant role in regulating apoptosis and autophagy [27, 28]. Research shows that STAT3/bcl-2 signaling is associated with the induction of autophagy in GBM [29]. Furthermore, we found that overexpressing RPL22L1 increased the expression of inhibitory apoptosis factor bcl-2. Treated with STAT3 inhibitor-Stattic and combined application with TMZ, the expression of bcl-2 in cells overexpressing RPL22L1 was decreased. Moreover, as we all known that ERK is upstream of STAT3 [30]. Besides, our team previously found that RPL22L1 activated ERK pathway independently of MAPK, promoting hepatocellular carcinoma (HCC) malignancy [31]. This potential mechanism needs to be further verified and studied. Inhibition of STAT3 is an efficient therapeutic to overcome TMZ resistance in GBM, and could be the next promising compound leading to survival prolongation [32]. Our data demonstrate that RPL22L1 affects the efficacy of TMZ by activating STAT3 in GBM, inhibition of STAT3 can reverse the TMZ resistance caused by RPL22L1. The combination of Stattic and TMZ has a better therapeutic effect of GBM with high RPL22L1 expression.

In conclusion, we verified that RPL22L1 promotes mesenchymal phenotype, invasiveness and GBM progression by activating EGFR/STAT3. It can be used as a promising indicator of STAT3 inhibition combined therapy for GBM, patients with high expression of RPL22L1 can benefit from the combination therapy.

## Materials and methods

### Ethics statement

All research performed was approved by the Ethics Review Board of Harbin Medical University and in accordance with the principles expressed at the Helsinki Declaration. Written informed consent of all participants was obtained. Animal experiments were performed according to Health guidelines of Harbin Medical University Institutional Animal Use and Care Committee.

### Tissue microarrays

Tissue microarrays (TMAs) HBraG125PG01, HBra-Gli060PG-01 (179 human glioma brain tissues and 6 normal brain tissues) and HBra-Gli080PG-01 (32 human GBM brain tissues and 3 normal brain tissues) were purchased from Outdo Biotech CO. Ltd. (Shanghai, China).

### Clinical tissue specimens

6 pairs of GBM and adjacent tissues (1.5 cm from the tumor tissue) were collected in the Department of Neurosurgery, the Harbin Medical University Cancer Hospital. Another 7 cases of GBM pathological sections were obtained from the Department of Pathology, the Harbin Medical University Cancer Hospital. All patients have signed informed consent forms.

### Bioinformatics data analysis

The RNA-seq data and corresponding clinical information of 167 patients with GBM and 5 normal tissues were downloaded from The Cancer Genome Atlas (TCGA) data portal (http://cancergenome.nih.gov/). The GSE4290, GSE30338, GSE7696 datasets were downloaded from the GENE EXPRESSION OMNIBUS (GEO) database (http://www.ncbi.nlm.nih.gov/geo). We also analyzed GBM data through the Oncomine platform (https://www.oncomine.org) and GEO2R platform (https://www.ncbi.nlm.nih.gov/geo/geo2r/).

### Cell lines and cell culture

Human GBM cell lines A-172 (CRL-1620^TM^), LN-229 (CRL-2611^TM^) and T98G (CRL-1690^TM^) were obtained from the American Type Culture Collection (ATCC, Manassas, VA). U251 (CL-0237^TM^) were kindly provided by Procell Life Science & Technology Co., Ltd. All cell lines were identified by STR (Genetic Testing Biotechnology, Beijing, China). Cells were cultured in Dulbecco’s Modified Eagle’s Medium (DMEM, Invitrogen) media supplemented with 10% fetal bovine serum (Gibco, USA) at 37 °C in a humidified atmosphere with 5% CO_2_ and were tested negative for mycoplasma contamination.

### Establishment of stable cell lines

The RPL22L1-GFP and the control lentivirus, RPL22L1-shRNA and negative control lentivirus were all purchased from Hanbio Technology Co., Ltd (Shanghai, China). GBM cell lines were infected with different lentiviruses vectors according to the manufacturer’s instructions. After 48–72 h of transfection, the fluorescence expression was observed under a fluorescence microscope, and puromycin screening was used to obtain stable cell lines for subsequent tests. Western Blot was used to verify the transfection efficiency.

### Protein extraction and Western Blot

Total proteins of GBM cells and tissues were extracted by prechilled RIPA buffer (Thermo Fisher Scientific, MA, USA) with proteinase and phosphatase inhibitor. Western Blot analysis was performed according to the standard protocol. Briefly, proteins were subjected to electrophoresis on 10/12.5% SDS-PAGE and transferred onto PVDF membranes (Millipore, Billerica, MA, USA). The PVDF membranes were blocked with 5% BSA (Amresco Inc., Washington state, USA) and were incubated with primary antibodies (Supplementary Table 1) overnight at 4 °C. Horseradish peroxidase (HRP)-labeled secondary antibodies (ZSGB-bio, Beijing, China) were incubated at room temperature for 1 h. The protein bands were visualized using the Enhanced Chemiluminescent (ECL) (Xin Sime, Suzhou, China) and Imaging Analysis System (Bio-Rad, California, USA). Uncropped Western Blots are shown in Original Data File.

### Cell proliferation analysis

Cells (5 × 10^3^/well) were seeded in 96-well plates for 5 days, and MTS (3-(4,5-dimethylthiazol-2-yl)-5-(3-carboxymethoxyphenyl)-2-(4-sulfophenyl)-2H-tetrazolium, inner salt) assay (G5421, Promega, America) and CCK8 (Cell Counting Kit-8) assay were performed according to the manufacturer’s protocol from the 1st day to the 5th day at the same time point. All experiments were performed three times independently.

### Wound healing, migration and invasion assay

In the wound healing experiment, the cells were seeded into 6-well plates and cultured until the confluence reached 80-90%. Wound healing was observed under the microscope after the wound was made with a 10 μl pipette tip. Corning chambers (Corning, MA, USA) with or without matrigel were used for the cell invasion and migration assays. All experimental procedures followed the manufacturer’s instructions. Briefly, the cells in serum-free medium were added to the upper chamber. The medium containing 20% fetal bovine serum as a chemical attractant was placed in the lower chamber. After 48 h of incubation at 37 °C, cells were fixed with 4% paraformaldehyde and stained with Hematoxylin–eosin (H&E) staining. The average number of cells was randomly selected from more than five microscope fields per chamber. All experiments were repeated three times independently.

### Immunofluorescence (IF) staining

Cells were seeded on cell slides (WHB-24-CS, Shanghai, China) in 24-well plates. Standard procedures were used for cell staining. Primary antibodies were diluted in 1% BSA in PBS (Supplementary Table 1). After overnight incubation at 4 °C, the cells were washed three times with PBS and incubated with FITC-labeled anti-IgG antibodies (Alexa Fluor 488 and 594, Thermo Fisher) for 1 h at room temperature. The DNA was stained with DAPI (4’-6-diamidino-2-phenylindole) (Sigma, USA) and visualized with a fluorescence microscope (Nikon C2, Tokyo, Japan) and a laser scanning confocal microscope (LSCM) (ZEISS LSM700, Germany).

### Xenograft model in vivo

Four-week-old female athymic BALB/c nude mice were purchased from Beijing Vital River Laboratory Animal Technology Co., Ltd. (Beijing, China). A total of 3 × 10^5^ cells (LN229-Vec/LN229-RPL22L1 and U251-NC/U251-shRPL22L1) per mouse were stereotactically injected into the brain. Mice bearing GBM that exhibited obvious weight loss ( ≥ 20% before the experiment) or onset of significant neurologic symptoms, such as seizures, impaired balance, and hemiplegia were considered in a moribund condition. When these symptoms were identified, mice were euthanized, the survival times were recorded and the brain tissues were obtained for further H&E and IHC assays.

For subcutaneous xenograft model, the mice were randomly divided into 6 groups (*n* = 5), 1 × 10^7^ cells (LN229-Vec/LN229-RPL22L1) were subcutaneously injected into the right flank of each nude mouse. In this animal experiment, we estimate that the sample size is less than 100 nude mice. After about 8 days post injection, all mice had developed subcutaneous tumors. The tumor size was measured with a caliper every other day, and the tumor volume was calculated according to the formula: volume = length × width × width/2. Meanwhile, mice were treated by intraperitoneal injection of prescribed dose of drugs and sacrificed when tumor volume reached about 1 cm^3^. All procedures were approved by the Committee on the Ethics of Animal Experiments of Harbin Medical University. For animal studies, we follow the principle of double blindness randomization.

### H&E and IHC staining

For H&E staining, slides were deparaffinized, hydrated, stained with hematoxylin for 8 min, and stained in eosin solution 30 s, then fixed with xylene, covered with neutral resin, observed under microscope.

For IHC staining, paraffin-embedded tumor specimens were cut into 5 mm thick sections and then dewaxed and rehydrated. Tissue slides were routinely treated with 3% H_2_O_2_ for 10 min at room temperature for antigen retrieval. After quenching for endogenous peroxidase and blocking with goat serum, sections were incubated sequentially with primary antibodies (Supplementary Table 1) and HPR-linked secondary antibodies. Sections were covered with diaminobenzidine (DAB) for visualizing the staining, and then counterstained with hematoxylin before being examined using a microscope.

### Colony formation assay

500 cells were planted in each well of 6-well plates and incubated with different treatments for 14 days. Then cells were fixed with 4% paraformaldehyde and stained with Giemsa agents.

### Flow cytometry analysis of cell apoptosis

1 × 10^6^ cells were collected for each group and stained with PI and Annexin V-FITC according to the manufacturer’s instructions (BD Biosciences, USA). The apoptosis rates were measured by flow cytometry (BD FACSCanto II, USA).

### TUNEL assay

Cells were fixed in 4% paraformaldehyde for 15 min and then stained with the In Situ Fluorescein TUNEL Cell Death Apoptosis Detection Kit (TransGen Biotech, Beijing) according to the manufacturer’s protocol.

### Statistical analysis

Measurement data were expressed as mean ± SD, and the statistically significant differences between the groups were estimated by two-tailed independent Student’s *t*-test. The overall survival curves were used to describe the survival distributions, and the Log-rank test was applied for assessing statistical significance between different groups. The intensity of immunoreactivity on TMA was divided into 0–3 levels based on a consensus of the three investigators, staining score = staining intensity (0–3) × staining area ratio (0–100%). The stain of IHC were evaluated by Student’s *t*-test on the staining score to compare the RPL22L1 expression in GBM brain tissues and normal brain tissues. The χ^2^ test (Fisher’s exact test) was used to examine the association between RPL22L1 expression and clinicopathologic features. The Pearson correlation coefficient and Spearman correlation analysis were used to analyze the correlations between variables. A value of *P* < 0.05 was considered to be statistically significant. All statistical analyses were performed using GraphPad software version 7.0 (GraphPad Software, CA, USA) or IBM SPSS Statistics 23.0 (SPSS, Chicago, USA).

### Supplementary information

Supplementary Information

Original Data File

Reproducibility checklist

## Data Availability

The data in the current study are available from the corresponding authors upon reasonable request.
