# Supplementary material for: RPL22L1, a novel candidate oncogene promotes temozolomide resistance by activating STAT3 in glioblastoma
Source: Cell Death Dis. 2023 Nov 20;14(11):757. doi: 10.1038/s41419-023-06156-6 (PMC10662465; doi:10.1038/s41419-023-06156-6)
Supplement: Supplementary file 1 — Supplementary Information [file 41419_2023_6156_MOESM1_ESM.docx]

**RPL22L1, a Novel Candidate Oncogene Promotes Temozolomide Resistance by Activating STAT3 in Glioblastoma**

Yunping Chen^1,2,3#^, Yu Mu^1,2#^, Qing Guan^1,2#^, Chenlong Li^4^, Yangong Zhang^5^, Yinzhi Xu^1,2^, Chong Zhou^1,2^, Ying Guo^1,2^, Yanan Ma^1,2^, Meiqi Zhao^1,2^, Guohua Ji^1,2^, Peng Liu^1,2^, Donglin Sun^1,2^, Haiming Sun^1,2^, Nan Wu^1,2*^, Yan Jin^1,2*^

^1^Laboratory of Medical Genetics, Harbin Medical University, Harbin 150081, China

^2^Key laboratory of preservation of human genetic resources and disease control in China (Harbin Medical University), Ministry of Education, Harbin 150081, China

^3^Department of Sport Science and Health, Harbin Sport University, Harbin, 150008, China.

^4^Department of Neurosurgery, Harbin Medical University Cancer Hospital, Harbin 150001, China

^5^Department of Neurosurgery, The Second Affiliated Hospital of Harbin Medical University, Harbin 150086, China

**Supplementary Figures S1 to S5**

**Supplementary Table S1**


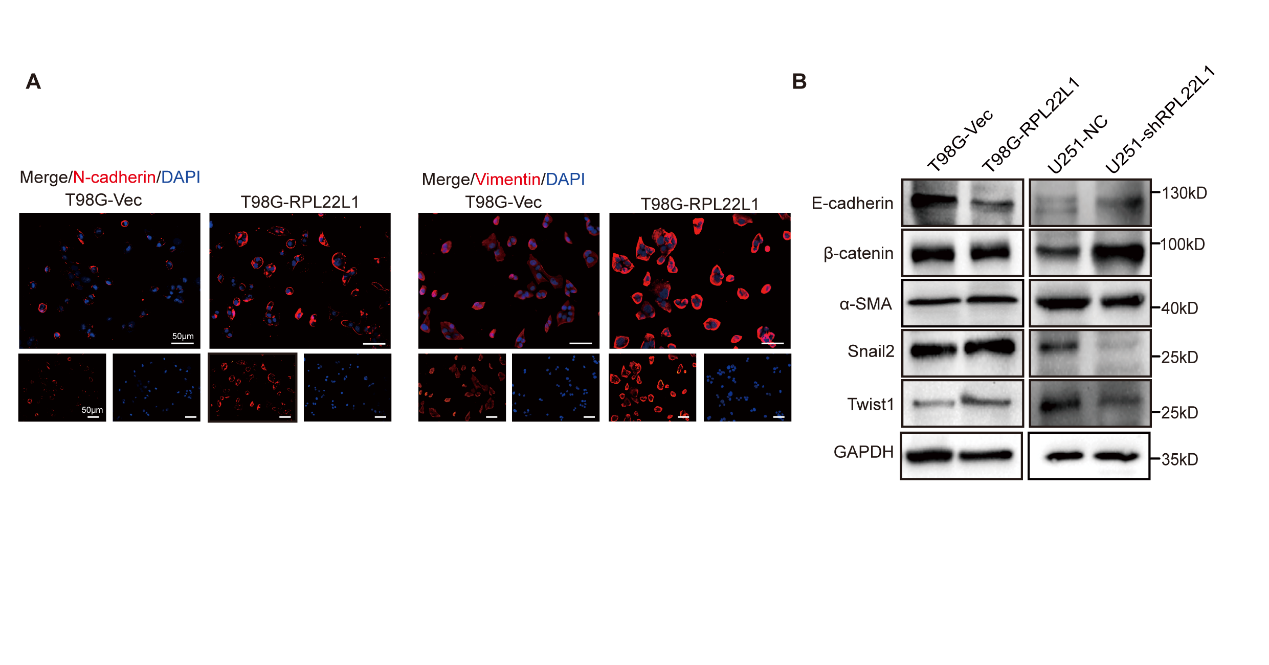


**Supplementary Fig. S1 RPL22L1 induces EMT in GBM cells.** **A** IF assay detected the expressions of N-cadherin and Vimentin protein (red: N-cadherin and Vimentin, blue: cell nucleus, magnification×200, scale bar=50μm). **B** The expressions of E-cadherin, β-catenin, α-SMA, Snail2 and Twist1 in GBM cells were detected by Western Blot, GAPDH was used as the internal control.

**
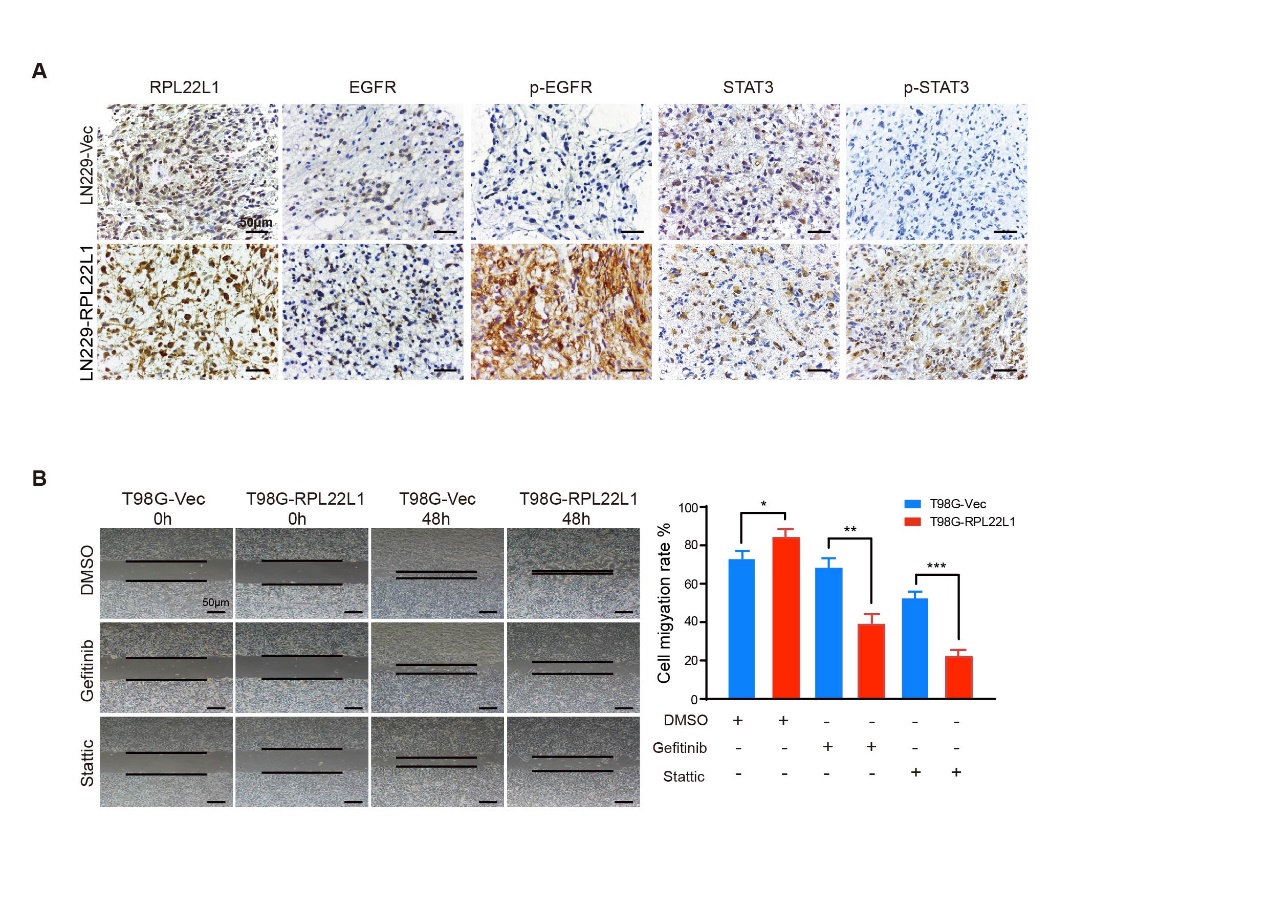
**

**Supplementary Fig. S2.** **RPL22L1 activates the EGFR/STAT3 pathway to promote the progression of GBM. A** IHC staining of RPL22L1, EGFR, p-EGFR, STAT3 and p-STAT3 in tumor sections of mice orthotopically xenografted with LN229-RPL22L1/LN229-Vec cells (magnification×400, scale bar=50μm). **B** T98G-RPL22L1/T98G-Vec cells were treated with Gefitinib (10μmol/L) and Stattic (5μmol/L), DMSO was used as the negative control. Wound-healing assay was used to detect the effects of Gefitinib or Stattic on the migration of RPL22L1 overexpressing cells (left, magnification×40, scale bar=50μm). All data were shown as mean±SD of three independent experiments (right, **P*<0.05, ***P*<0.01; ****P*<0.001, Student’s *t* test).

**
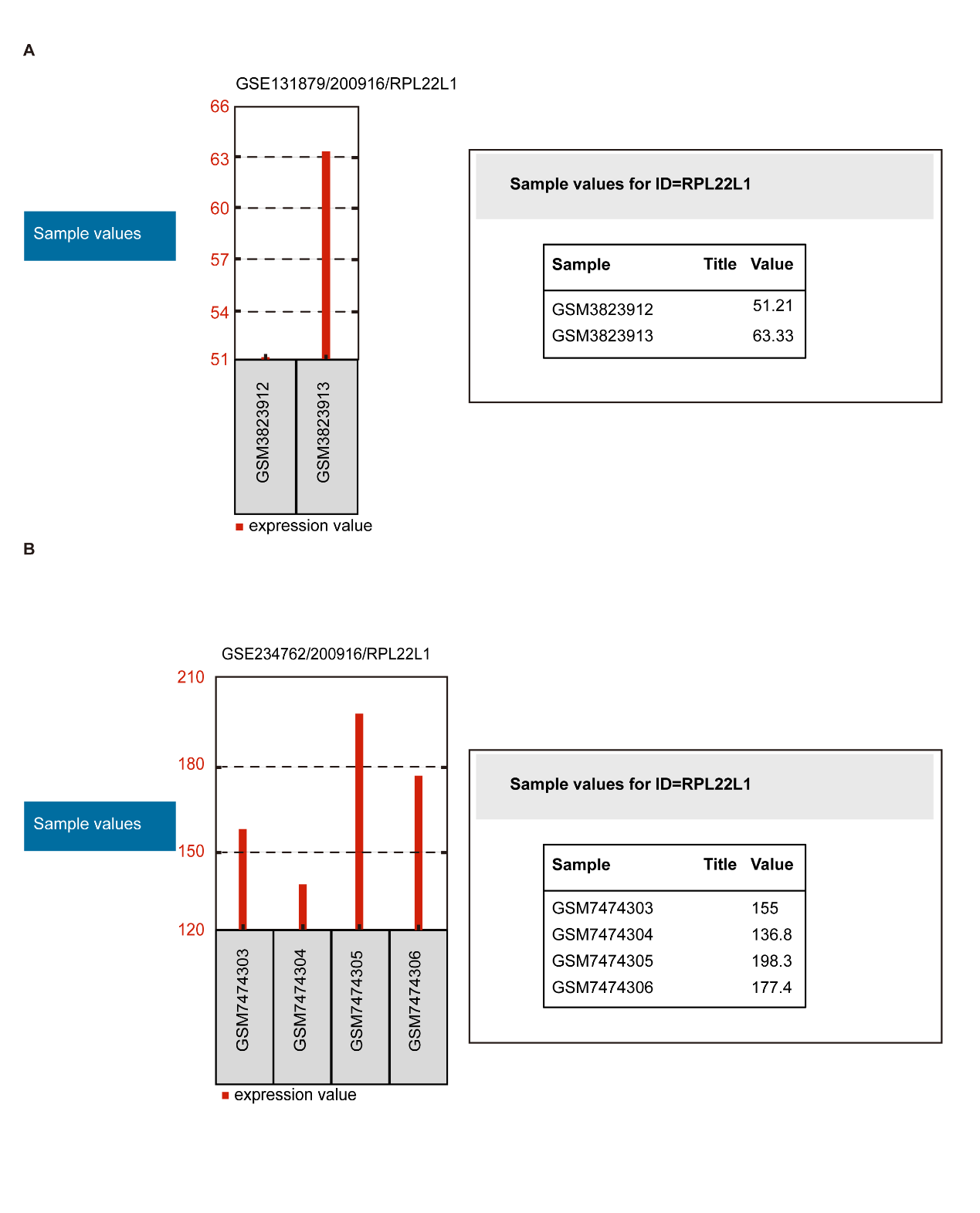
****Supplementary Fig. S3**. **RPL22L1 is increased in GBM resistant cell lines.** RPL22L1 mRNA levels in GBM resistant cell lines were analyzed in GEO datasets (GSE131879, GSE234762) through GEO2R platform, (<https://www.ncbi.nlm.nih.gov/geo/geo2r/>). **A** The gene expressional profiles between A172 glioma cells and the derived TMZ-resistance cells. Platform: GPL20795 HiSeq X Ten (Homo sapiens). GEO Accession: GSM3823912, Title: A172, Source name: GBM, Cell type: parental, Treatment: without TMZ, Cell line: A172. GEO Accession: GSM3823913, Title: A172R6, Source name: GBM, Cell type: resistance, Treatment: with TMZ, Cell line: A172-r#6. **B** Gene expression profiling of TMZ-resistant GBM. Platform: GPL24676 Illumina NovaSeq 6000 (Homo sapiens). GEO Accession: GSM7474303, Title: GBM8401 cells, Source name: brain, Tissue: brain, Cell line: GBM8401, Cell type: Human brain GBM multiforme, Treatment: parental. GEO Accession: GSM7474304, Title: GBM8401 cells, Source name: brain, Tissue: brain, Cell line: GBM8401, Cell type: Human brain GBM multiforme, Treatment: parental. GEO Accession: GSM7474305, Title: GBM8401. TMZ-resistant cells, Source name: brain, Tissue: brain, Cell line: GBM8401 TMZ-resistant cells, Cell type: Human brain GBM multiforme, Treatment: TMZ-resistant. GEO Accession: GSM7474306, Title: GBM8401. TMZ-resistant cells, Source name: brain, Tissue: brain, Cell line: GBM8401 TMZ-resistant cells, Cell type: Human brain GBM multiforme, Treatment: TMZ-resistant.

**S****upplementary Fig. S4. RPL22L1 promotes TMZ resistance in GBM resistant cell line. A** The expressions of endogenous RPL22L1 protein in LN229 and LN229R cells were detected by Western Blot, GAPDH was used as the internal reference. **B**
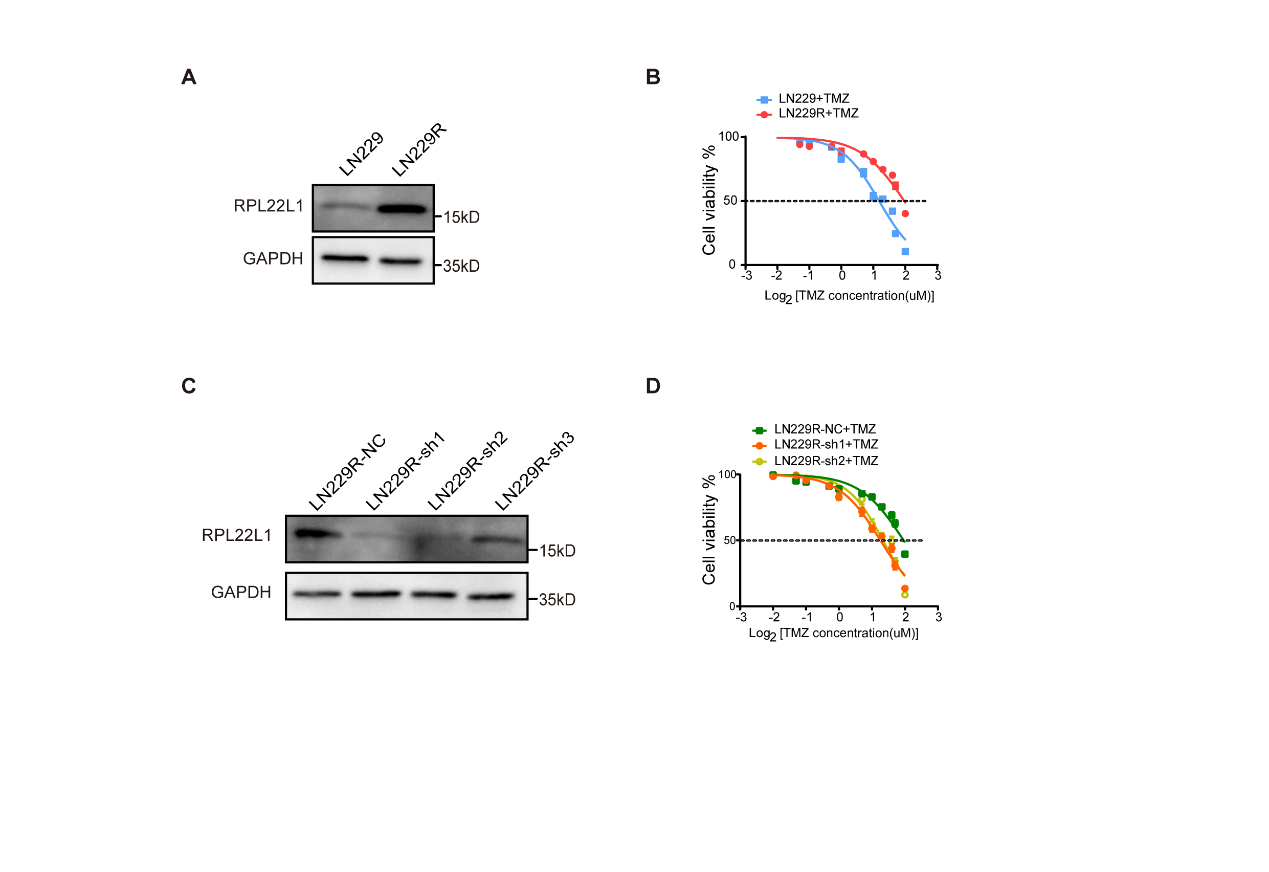
LN229R cells were treated with TMZ at the specified concentration for 48h and IC50 were determined by CCK8 method. **C** RPL22L1 expressions were efficiently knocked down by three targeted shRNAs (sh1, sh2 and sh3) in LN229R cells detected by Western Blot, NC served as negative control. **D**
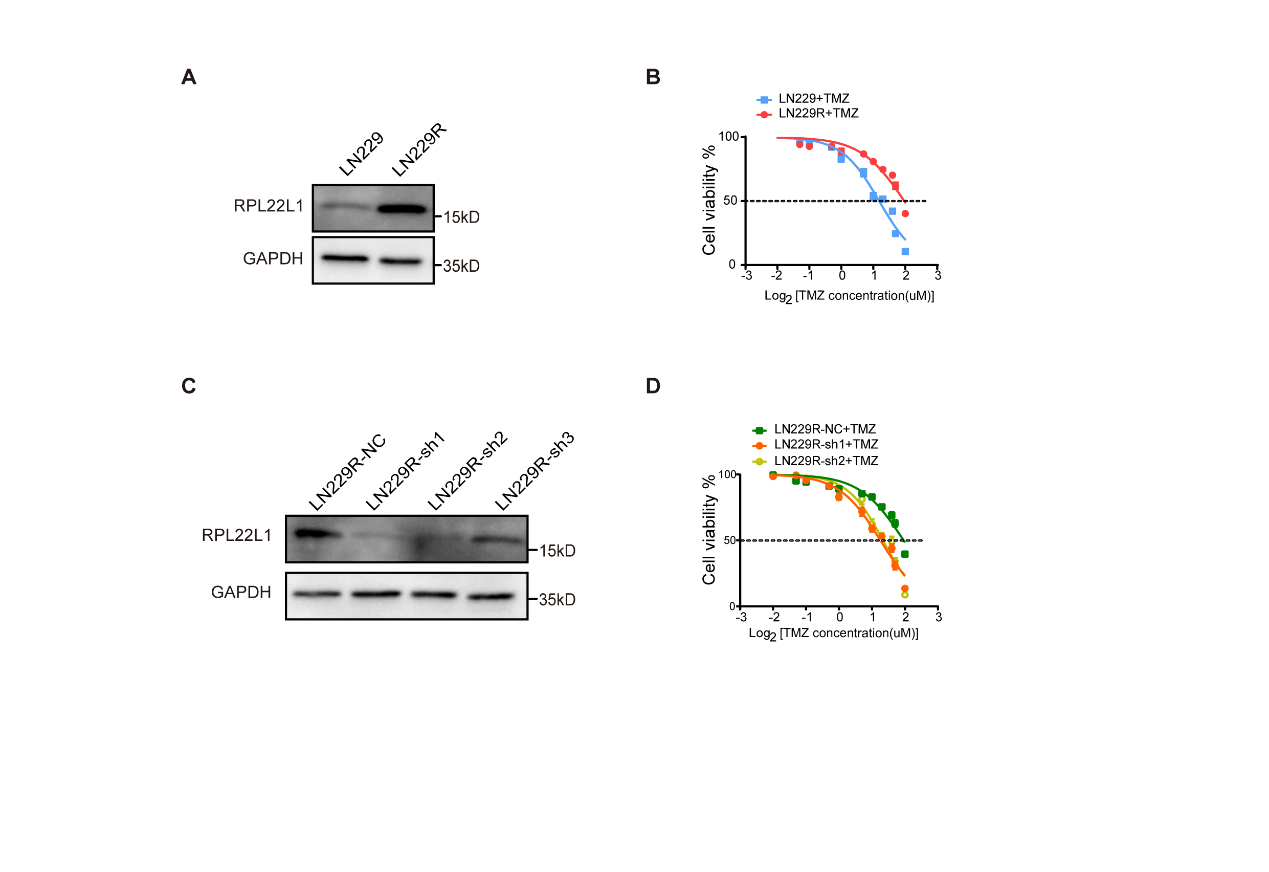
LN229R and LN229R-sh1, LN229R-sh2 cells were treated with TMZ at the specified concentration for 48h and IC50 were determined by CCK8 method.

**Supplementary Fig. S5. RPL22L1 mRNA expression level in GBM cell line**
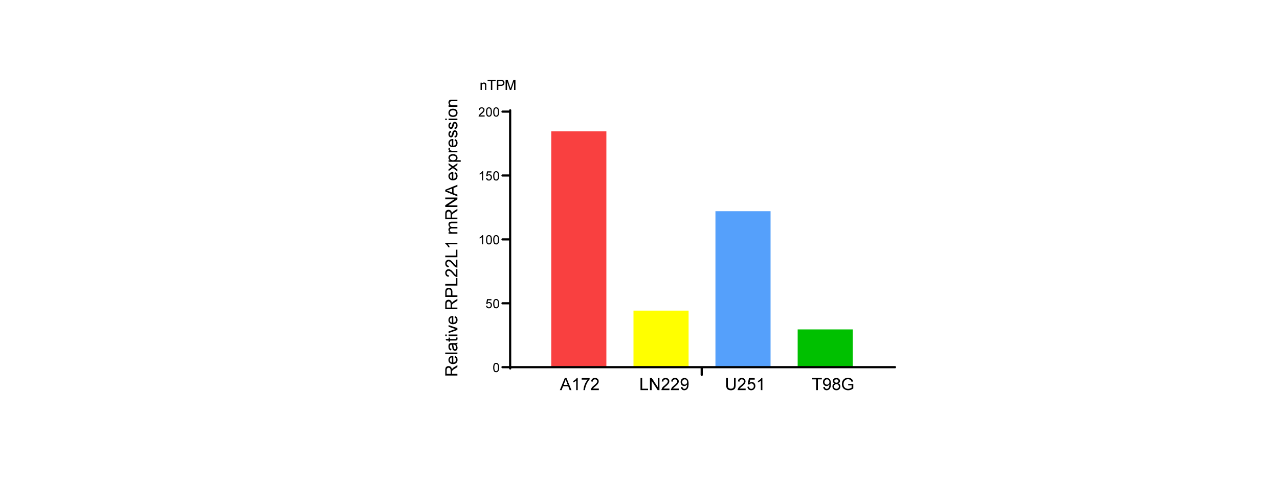
**s****.**

| **Antibody** | **Manufacturer** | **Catalogue numbers** | **Application** |
| --- | --- | --- | --- |
| RPL22L1  E-cadherin  β-catenin  N-cadherin  Snail2  Twist1  Vimentin  α-SMA  EGFR  p-EGFR  STAT3  p-STAT3  bcl-2  Bax  Cleaved-caspase-3  GAPDH | ABclonal  Proteintech  Proteintech  Proteintech  Proteintech  Proteintech  Proteintech  Proteintech  Santa  Cell Signaling Technology  Proteintech  Cell Signaling Technology  Proteintech  Cell Signaling Technology  Cell Signaling Technology  Proteintech | WG-03688, 9025-1  20874-1-AP  51067-2-AP  22018-1-AP  12129-1-AP  25465-1-AP  10366-1-AP  14395-1-AP  sc-373746  2236S  10253-2-AP  9145  12789-1-AP  5023S  9964S  60004-1-Ig | 1:500 for WB, 1:50 for IF, 1:100 for IHC  1:2000 for WB, 1:50 for IF,  1:2000 for WB  1:2000 for WB, 1:100 for IHC  1:2000 for WB  1:2000 for WB  1:2000 for WB, 1:50 for IF, 1:200 for IHC  1:2000 for WB  1:1000 for WB，1:100 for IHC  1:1000 for WB，1:50 for IHC  1:2000 for WB，1:100 for IHC  1:2000 for WB，1:50 for IHC  1:1000 for WB  1:1000 for WB  1:1000 for WB  1:3000 for WB |

**Supplementary Table S1. Primary antibodies used in the present study**
