# Supplementary figures and images for: RPL22L1, a novel candidate oncogene promotes temozolomide resistance by activating STAT3 in glioblastoma
Source: Cell Death Dis. 2023 Nov 20;14(11):757. doi: 10.1038/s41419-023-06156-6 (PMC10662465; doi:10.1038/s41419-023-06156-6)

**Original Western blots**

**
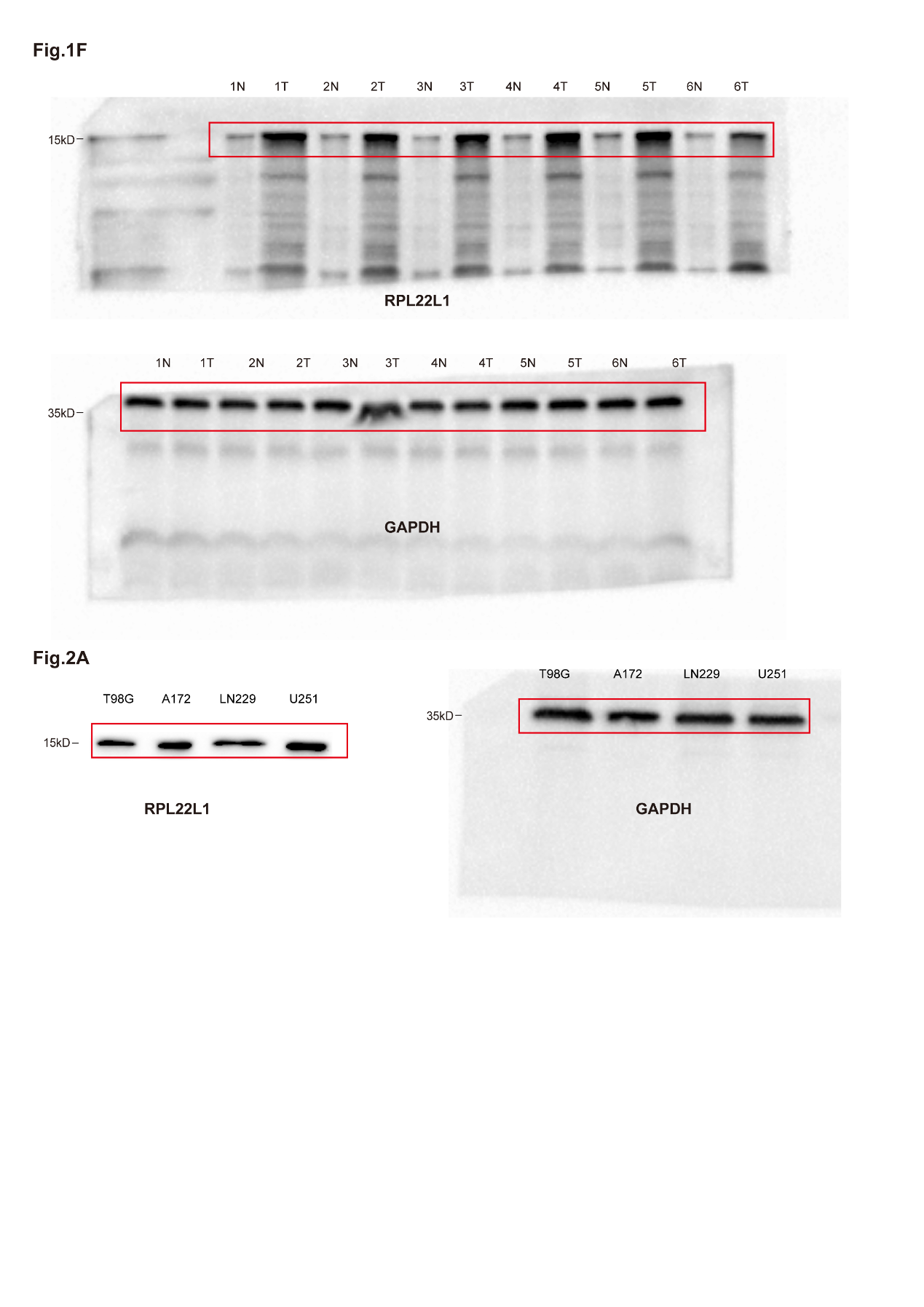
**


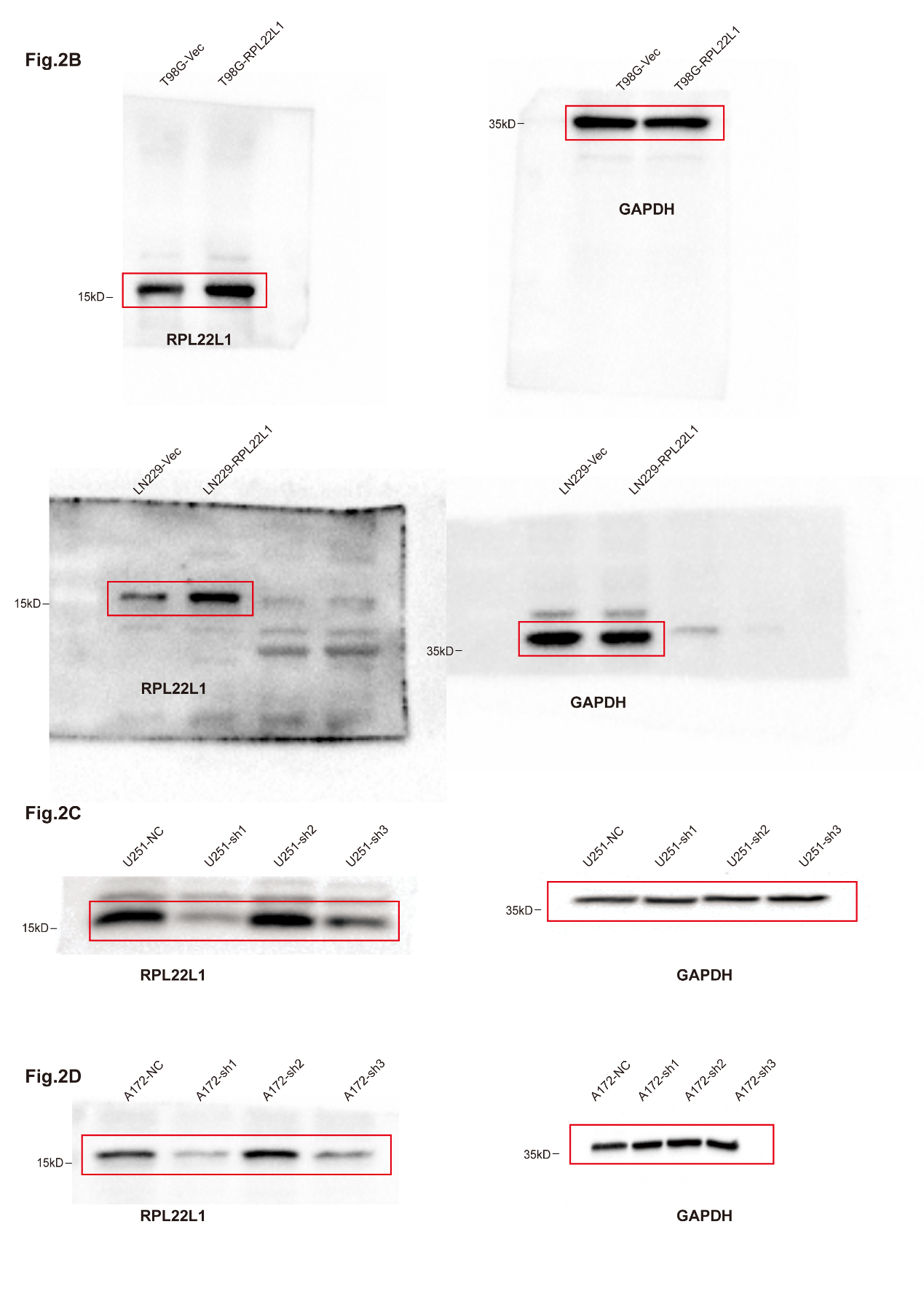


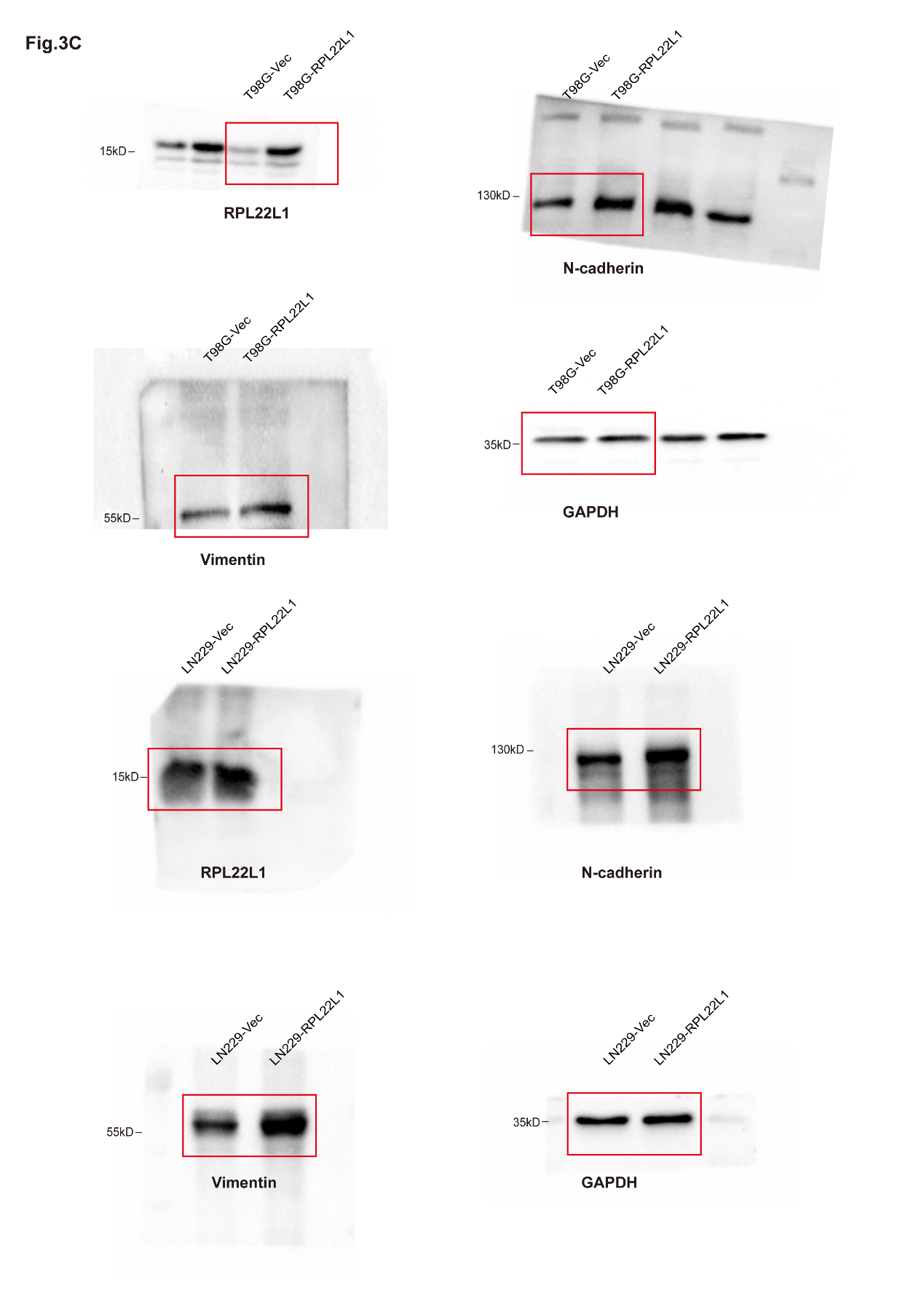


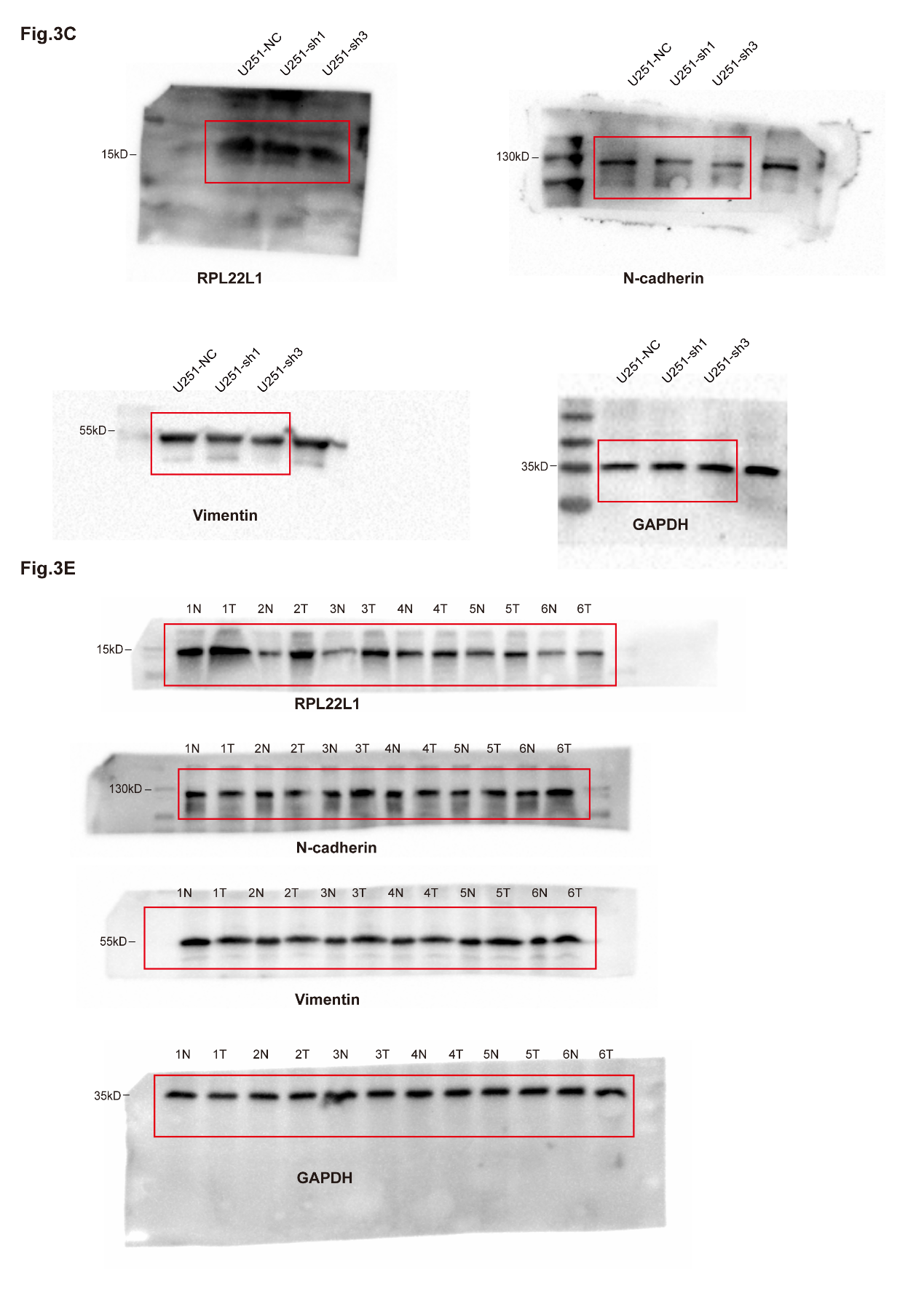


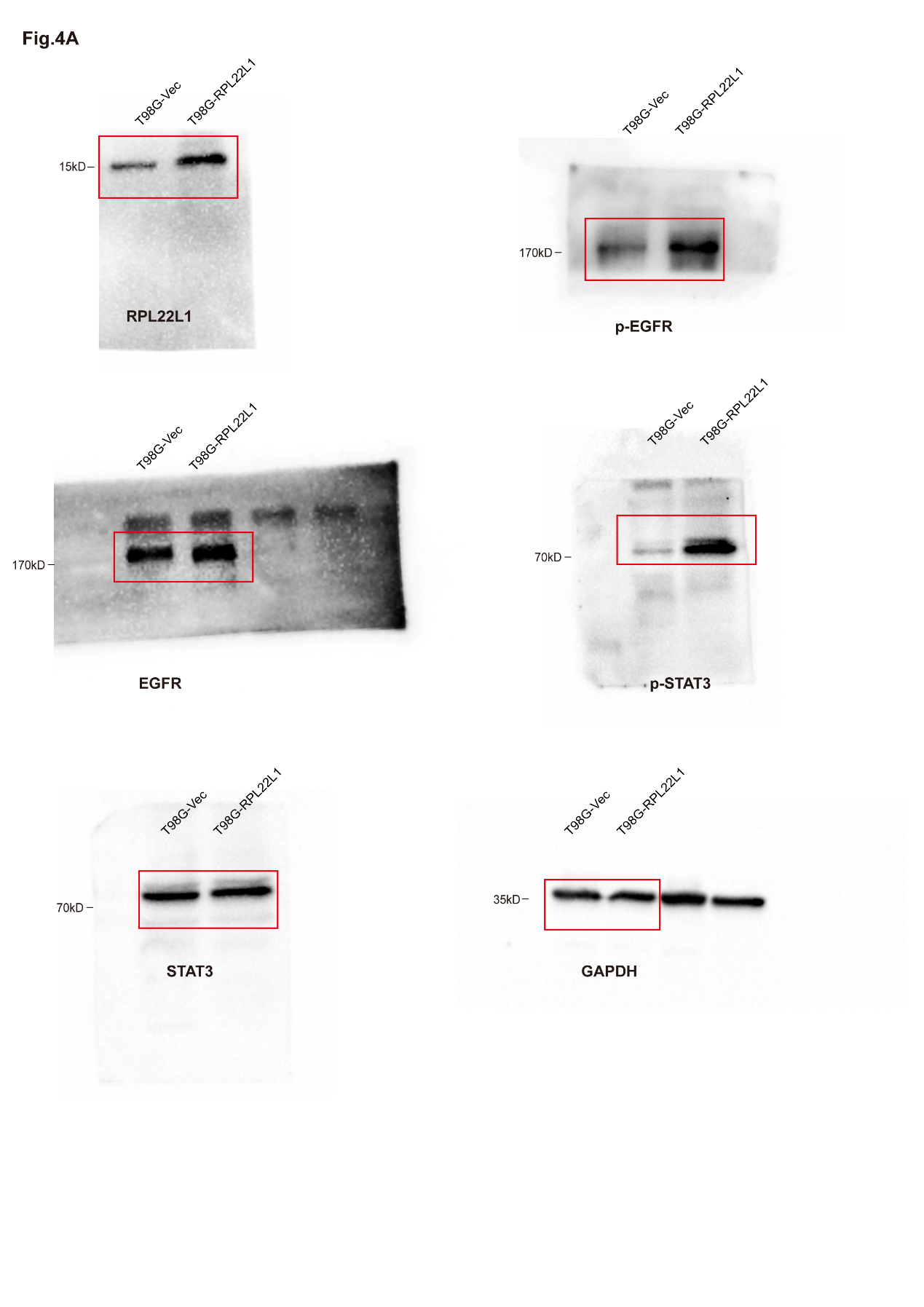


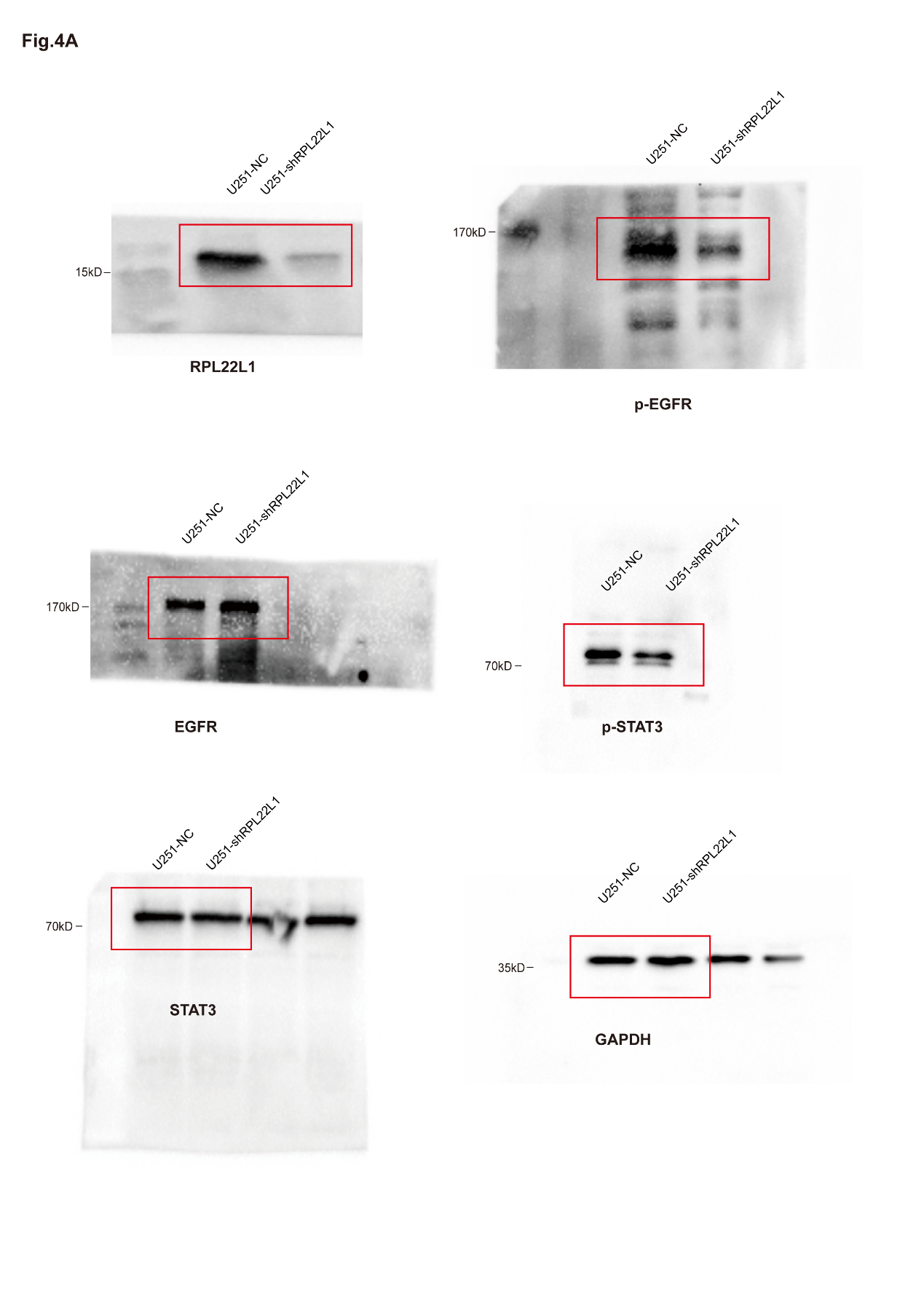


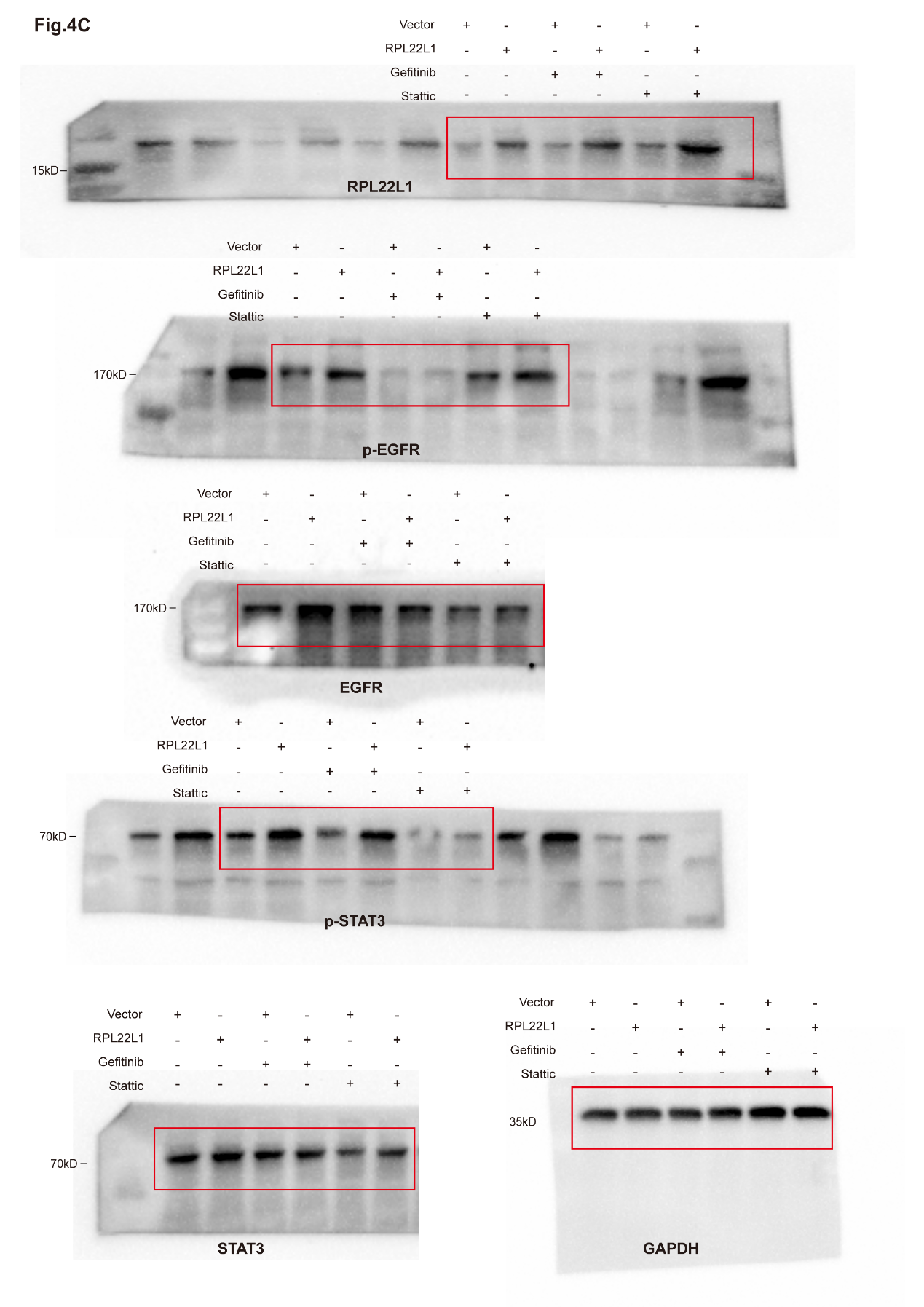


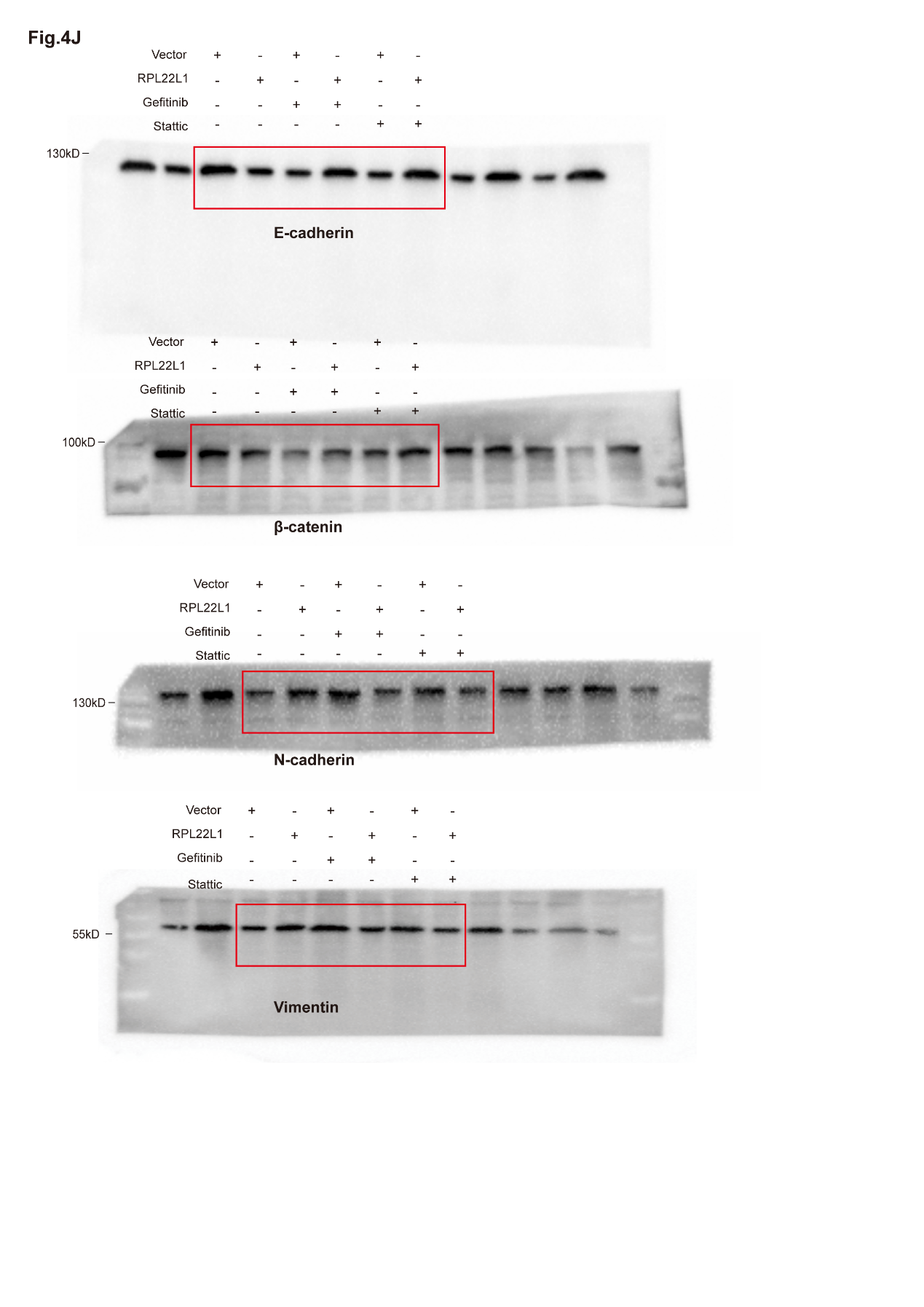


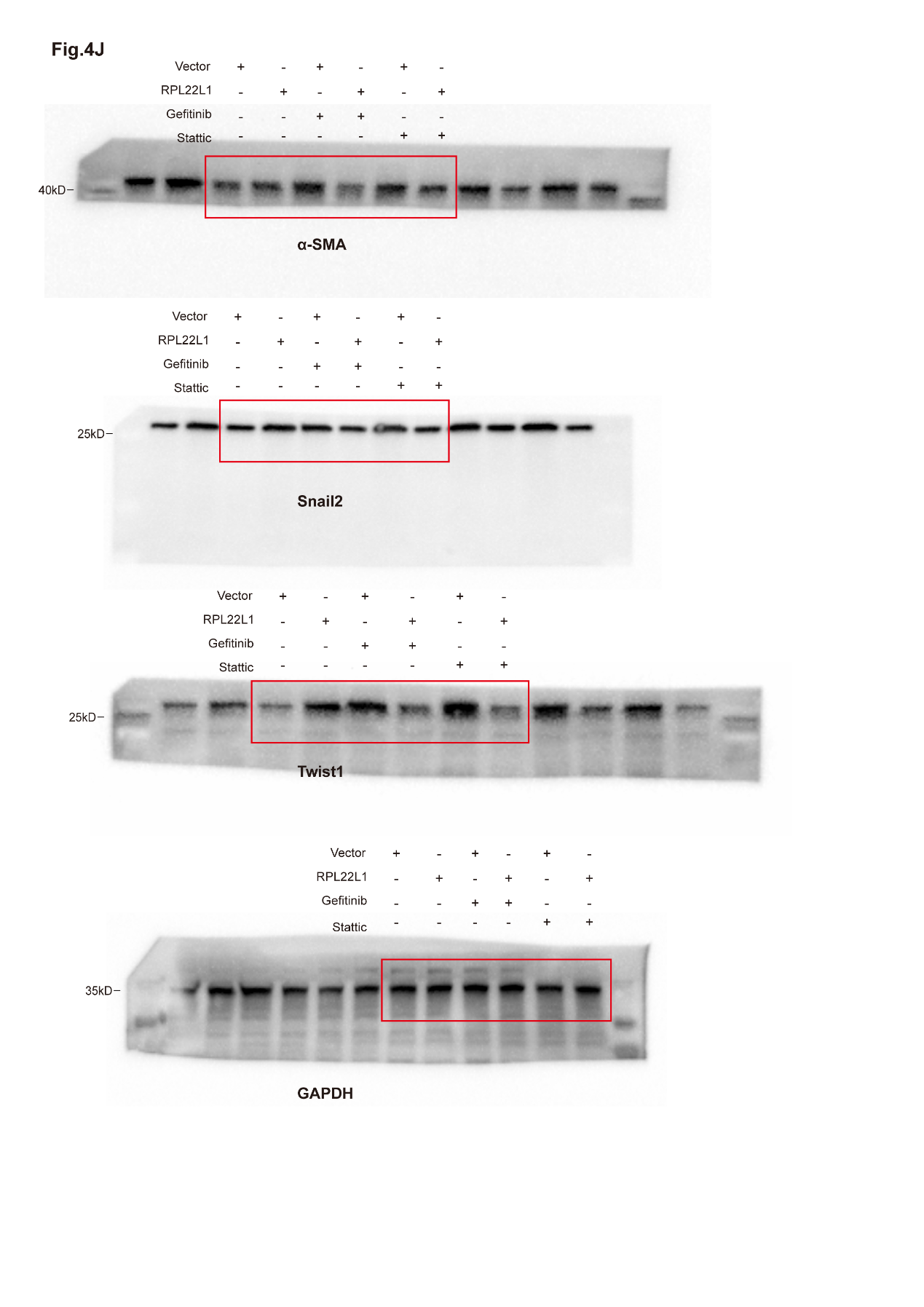


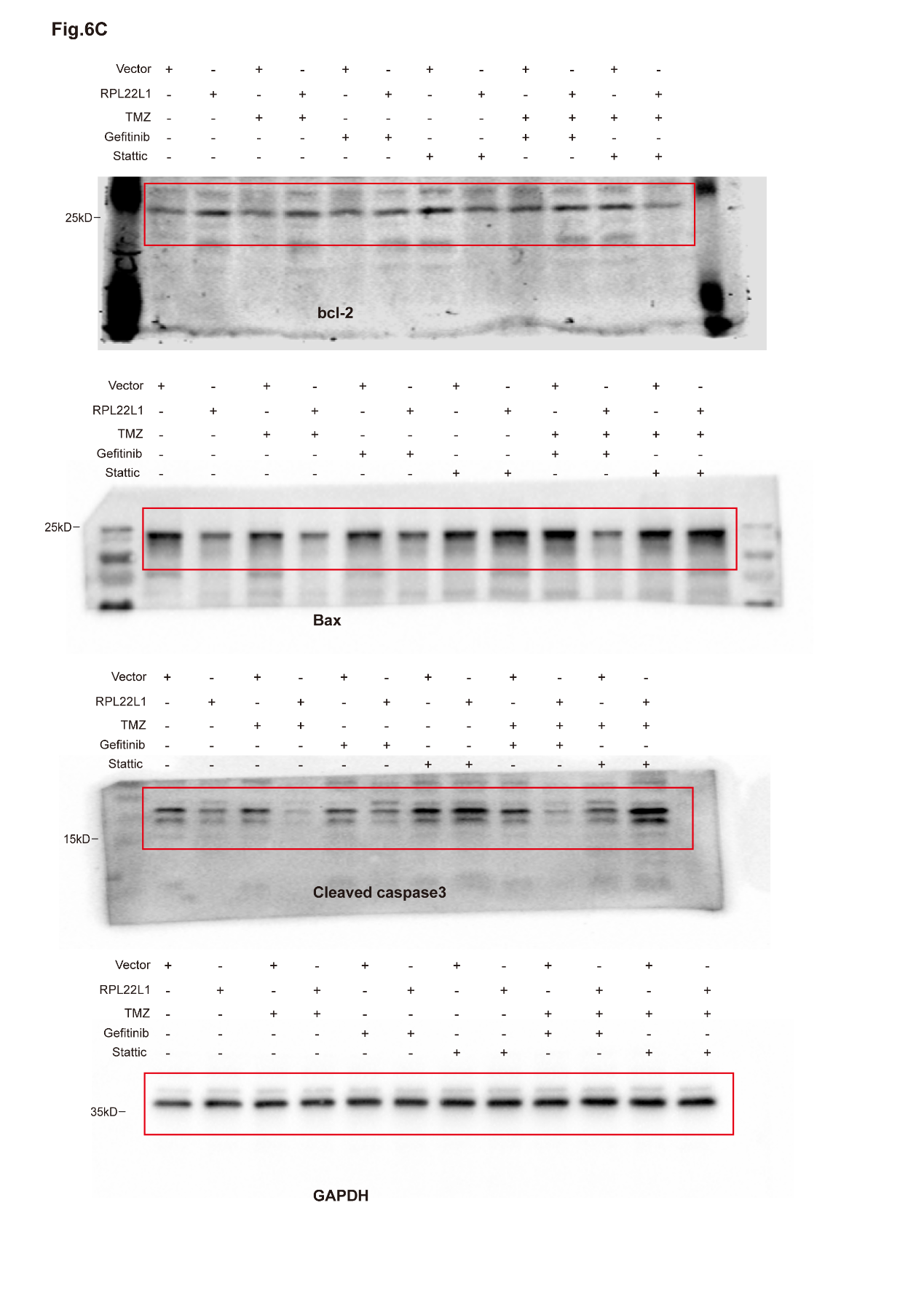


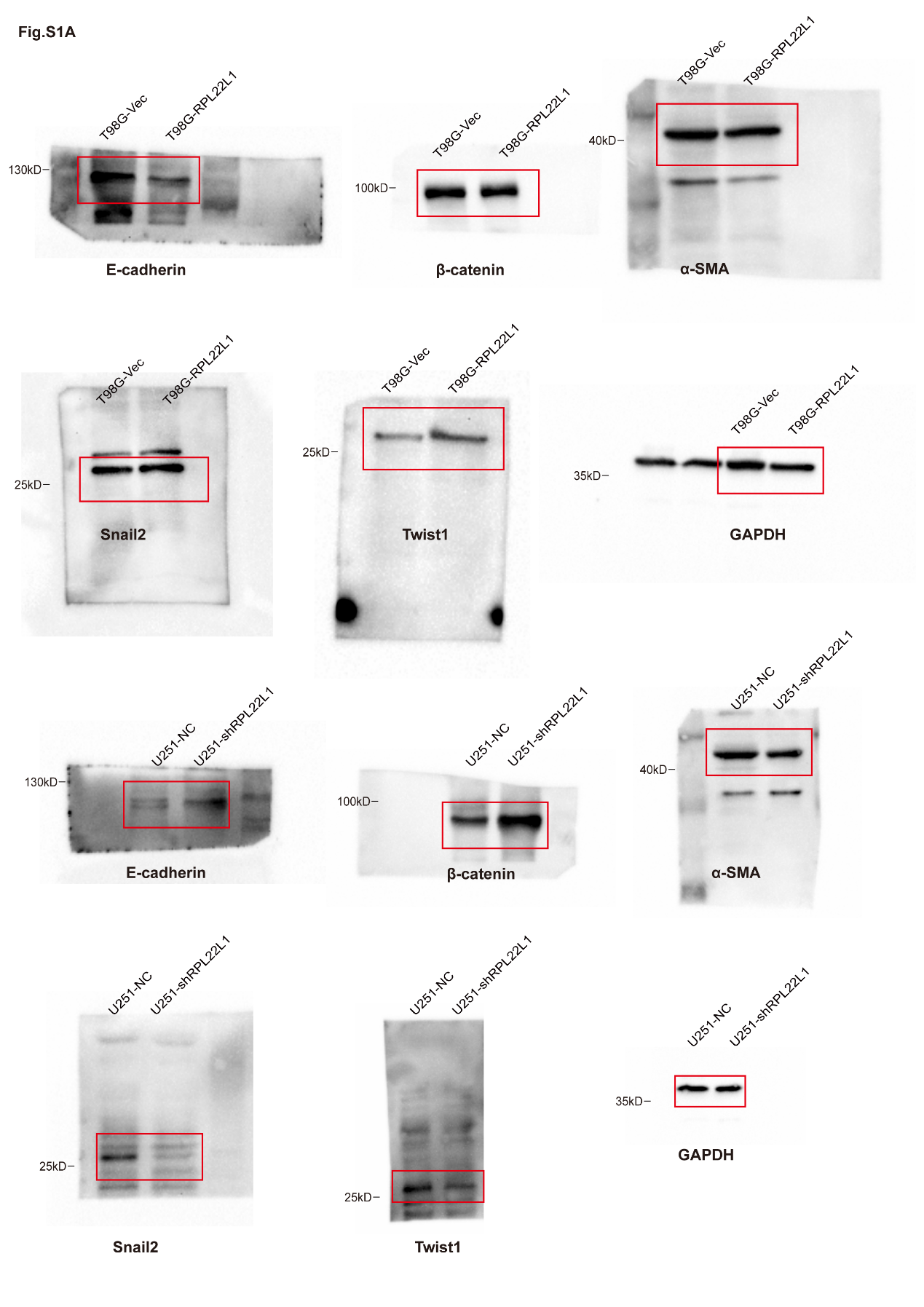


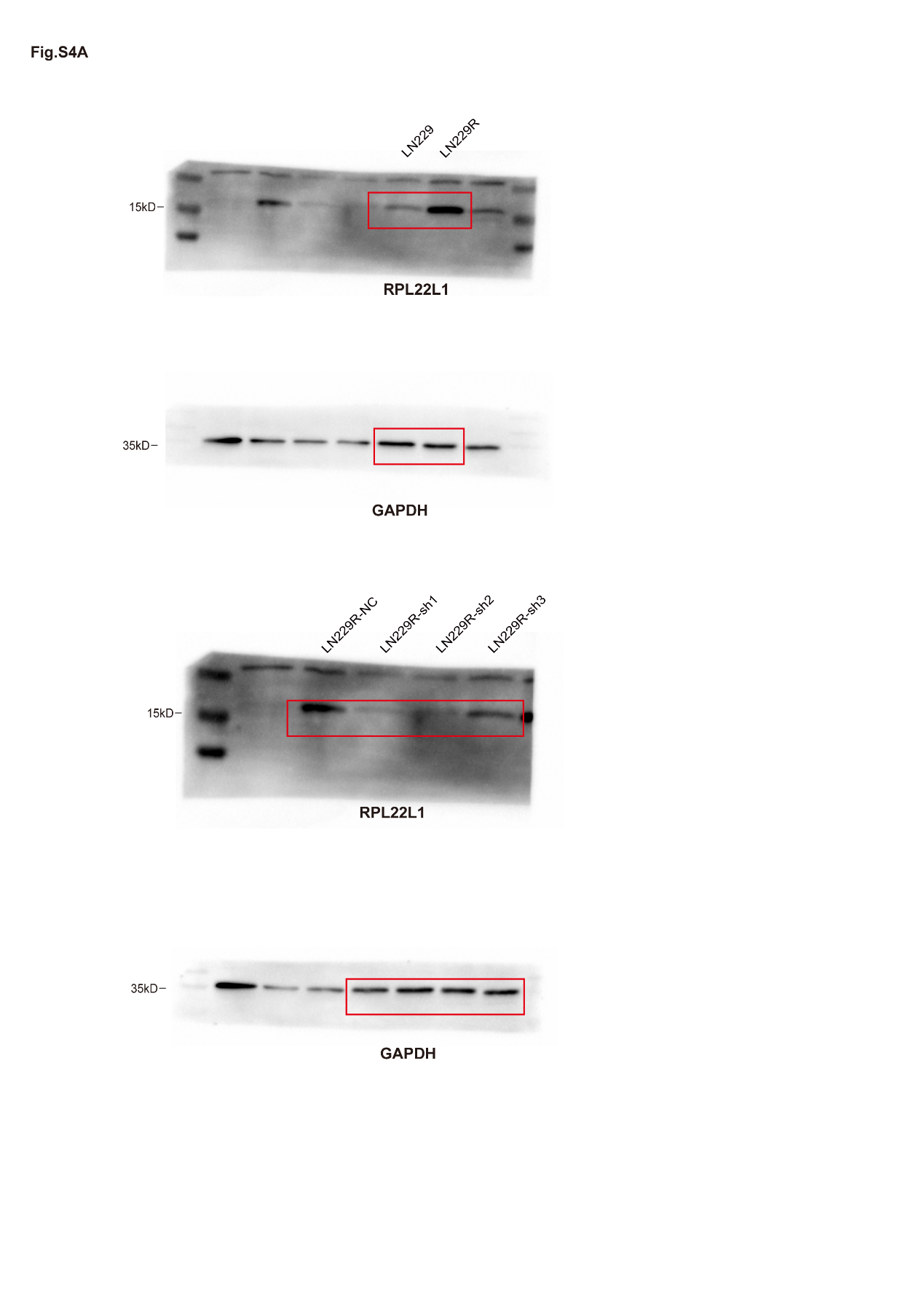

Supplement: Supplementary file 2 — Original Data File [file 41419_2023_6156_MOESM2_ESM.docx]
